# Selective Hydrolysis of Heterooligosaccharides by Poly(acrylate) Gel Catalysts

**DOI:** 10.1021/acscatal.4c04697

**Published:** 2024-10-30

**Authors:** Susanne Striegler

**Affiliations:** Department of Chemistry and Biochemistry, University of Arkansas, 345 North Campus Walk, Fayetteville, Arkansas 72701, United States

**Keywords:** carbohydrate, oligosaccharide, catalysis, glycosidic bond, nanogels, hydrolysis, biomimetic, selectivity

## Abstract

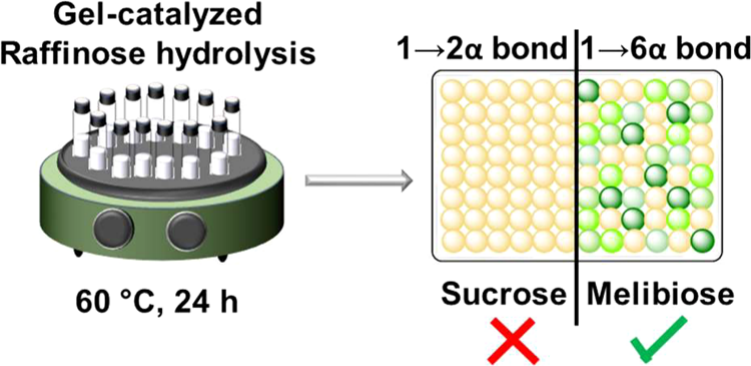

Natural glycoside hydrolases are distinguished by their ability to hydrolyze glycosidic bonds with high efficiency and selectivity. This feature is achieved through specific interactions in the active site during catalytic turnover and is not just facilitated by two catalytically active amino acids. Intrigued by these features, a biomimetic α-galactosidase mimic was developed using an empirical catalyst design. Starting with a library of 704 gels of which 250 have a unique composition synthesized from TEGDMA cross-linker and 7 selected monomers, 238 monomodal gels are evaluated for their ability to hydrolyze the 1→6 α-glycosidic bond in the disaccharide melibiose. Among those, 13 polyacrylate gels with the potential for high catalytic activity are identified using spectrophotometric screening assays based on Schiff bases formed with toluidine. The best-performing polyacrylate (gel A) was found to have a 1500-fold higher proficiency to hydrolyze the 1→6 α-glycosidic bond in melibiose over the 1→2 α-glycosidic bond in sucrose, translating to selective hydrolysis of the glycosidic linkages in the trisaccharide raffinose. The matrix of gel A is composed of 25 mol % TEGDMA cross-linker and equimolar amounts of cyclohexyl, butyl, and benzyl acrylate accounting for CH-π and hydrophobic interaction in the surrounding of a hydrolytic binuclear Cu(II) complex. The combined observations underline a paramount influence of matrix-stabilizing effects on the transition state of the hydrolysis of glycosidic bonds and may pave the way for the rapid development of catalysts transforming biomass.

## Introduction

**Chart 1:**
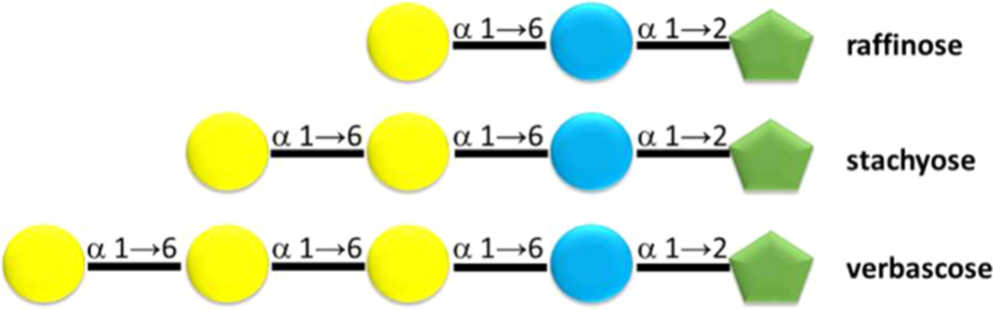
Oligosaccharides of the Raffinose Family

Many enzymes catalyze hydrolysis reactions to break down complex molecules into simpler entities. For example, proteases hydrolyze peptide bonds in proteins,^1−3^ nucleases break down phosphodiester bonds in nucleic acids,^4−6^ and glycosylases cleave off glycosidic bonds in carbohydrates and glycosides into simpler sugars.^7−9^ By contrast to proteins and other biomolecules, oligosaccharides can possess more than one type of bond among the glycosyl moieties and/or between glycosyl units and their aglycons.^10^ The most common linkages include α/β1→4 and 1→6 glycosidic bonds, but any other type is known and found in Nature. Due to the diversity of glycosidic linkages, the cleavage of saccharides is more complex than that in other compound classes. Consequently, most glycoside hydrolases have evolved to be very specific for certain substrates or particular glycosidic bonds. The observed enzyme specificity is due to the unique structure of its active site, which accommodates only selected substrates for binding and transformation. For example, the natural function of α-galactosidase (α-GAL E.C. 3.2.1.22) in the lysosome is the breakdown of glycolipids and glycoproteins containing terminal α-galactosyl moieties.^11^ The enzyme is also able to selectively cleave off the terminal galactose moieties in raffinose, stachyose, or verbascose by hydrolyzing the terminal 1→6 α-glycosidic bond(s), while the other bond, a 1→2 α-linkage between glucose and fructose, remains intact (Chart 1).

The intriguing selectivity and efficiency of enzymes prompted many attempts in biomimetic catalysis to achieve similar activity and features in man-made catalytic models over the past decades.^12−24^ However, the overall progress remains slow, and efficient biomimetic catalysts promoting glycoside hydrolase activity are scarce. Notable highlights of recent achievements in this regard include foldamer-based artificial glycosidases,^25^ molecularly imprinted nanoparticles,^26−28^ artificial metalloenzymes and peptides,^29−31^ and cross-linked micro- and nanogels targeting the hydrolysis of underivatized di- and oligosaccharides.^32−35^

Of particular importance in the context of further advancements in the field is a recently introduced empirical catalyst design combined with spectrophotometric screening assays.^34^ The method was designed to visualize the catalytic activity of gel catalysts on nonchromophore containing di- and oligosaccharides.^34,35^ In short, the procedure encompasses the synthesis of a large library of polyacrylate gels with a random monomer composition. Thereby, secondary catalysis-supporting matrix effects can be introduced into each gel. Each member of the library is initially screened for particle dispersity using dynamic light scattering. Monomodal gels are then selected for screening of their hydrolytic activity toward a disaccharide with the targeted glycosidic bond. The higher the hydrolytic activity of the original gel catalyst, the more monosaccharide is formed during hydrolysis and captured as a dark green Schiff base after treatment with toluidine in 96-well plate assays. This step narrows down a 100+ gel library to a few gels with the highest potential of hydrolytic activity toward the targeted glycosidic bond. In a final step, the selected gels are subjected to kinetic analysis with the targeted oligosaccharide to document their catalytic performance.

The previous proof-of-concept study used the disaccharide maltose for initial screening of gels for their ability to catalyze the hydrolysis of 1→4 α-glycosidic bonds. The remarkable activity of the identified gels to break down maltotriose was subsequently documented. The best-performing gel showed a catalytic proficiency (*k*_cat_/(K_M_ × *k*_non_)) of 2 × 10^6^ corresponding to a catalytic efficiency (*k*_cat_/*K*_M_) of 2.6 min^–1^ M^–1^.^34^ The catalytic performance was correlated to transition-state-stabilizing CH-π and hydrophobic interactions introduced by the embedded acrylate monomers. However, as the two glycosidic bonds in the hydrolyzed trisaccharide are identical, evidence for selective hydrolysis could not be obtained due to the design and focus of the previous examination. Intrigued by the two different glycosidic bonds in the trisaccharide raffinose and the efficiency of the empirical catalyst design, we set out here to develop, identify, and characterize gels with enzyme-like specificity upon hydrolyzing the terminal α-galactosyl moiety in raffinose.

## Results and Discussion

### Gel Synthesis

**Scheme 1:**
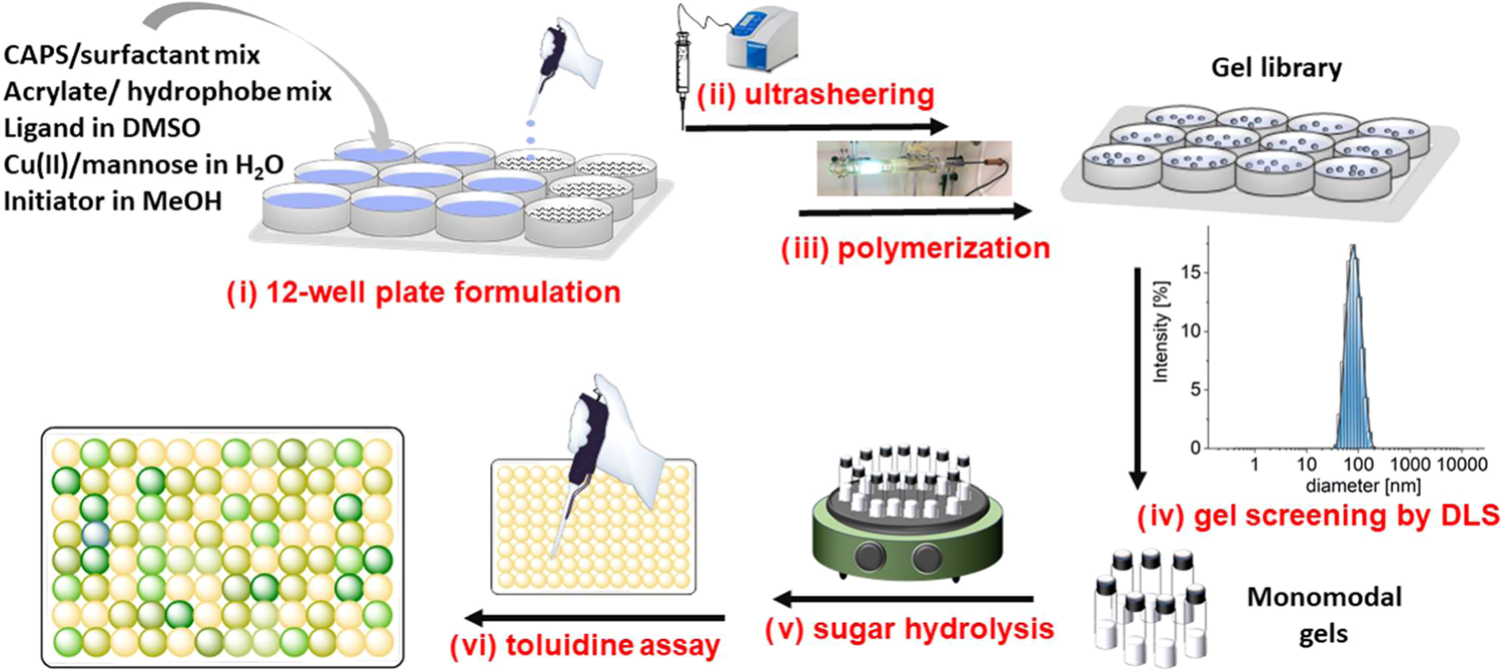
Synthesis of Polyacrylate Gels and Selection of Potential Catalysts for Hydrolysis of 1→6 α-Glycosidic Bonds

**Chart 2:**
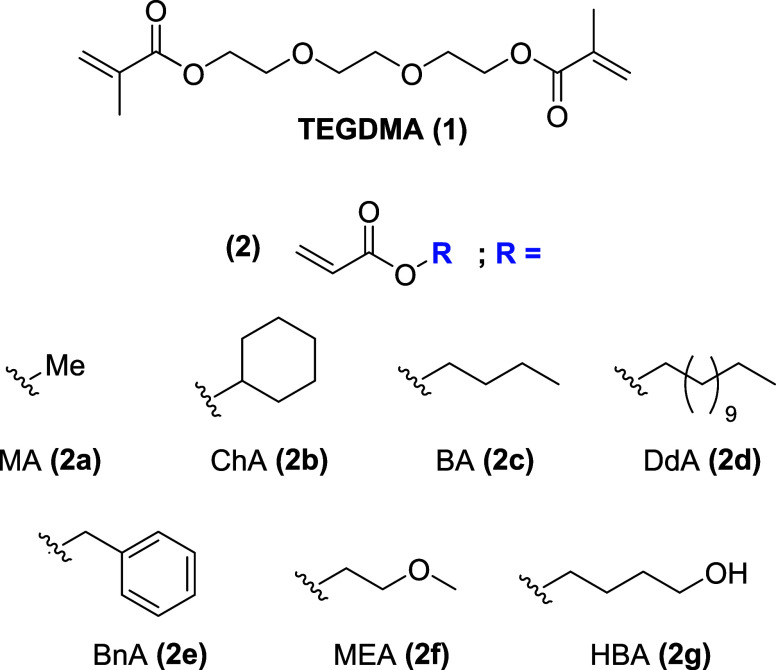
Structures of Acrylate Cross-Linker and Monomers

Initially, a library of 704 polyacrylate gels is synthesized, of which 250 have a unique composition containing TEGDMA (**1**) as a cross-linker and a systematically altered combination of 7 acrylate monomers (**2**) (Chart 2). The gels are synthesized in 12-well plates from miniemulsions by UV-light-initiated free radical polymerization over 30 min as described.^34,35^ Previous extensive studies based on gravimetric analyses established that the elaborated polymerization time ensures near-quantitative polyacrylate formation independently of the composition of the acrylate mixtures.^35^ The miniemulsions are obtained in each well by ultrasheering 2 mL of a CAPS-surfactant buffer solution, appropriate aliquots of acrylate-hydrophobe mixtures, and fixed aliquots of stock solutions for ligand VBbsdpo (**3**), Cu(II) acetate, mannose, and radical initiator (see Supporting Information and Scheme 1).^34,35^

Aside from the H-bond accepting interactions of the TEGDMA cross-linker, the monomers provide hydrophobic, H-bond-accepting and -donating as well as CH-π stacking interactions in the resulting matrix in addition to the H-bond-accepting properties of **1**. The monomer choice is inspired by the residues of amino acids typically found in the active site of glycosylases and influenced by previous results using the same strategy to identify gels for the hydrolysis of 1→4 α-glycosidic bonds in maltose and maltotriose.^34^ During the syntheses, the combined amount of matrix-forming acrylates per well is 0.35 mmol, of which 25 mol % are TEGDMA and 75 mol % are mixtures of up to 6 out of the 7 monomers. The smallest amount for each of the monomers **2** is arbitrarily set to 6.25 mol % (see the Supporting Information).

### Selection of Monomodal Gels

**Figure 1:**
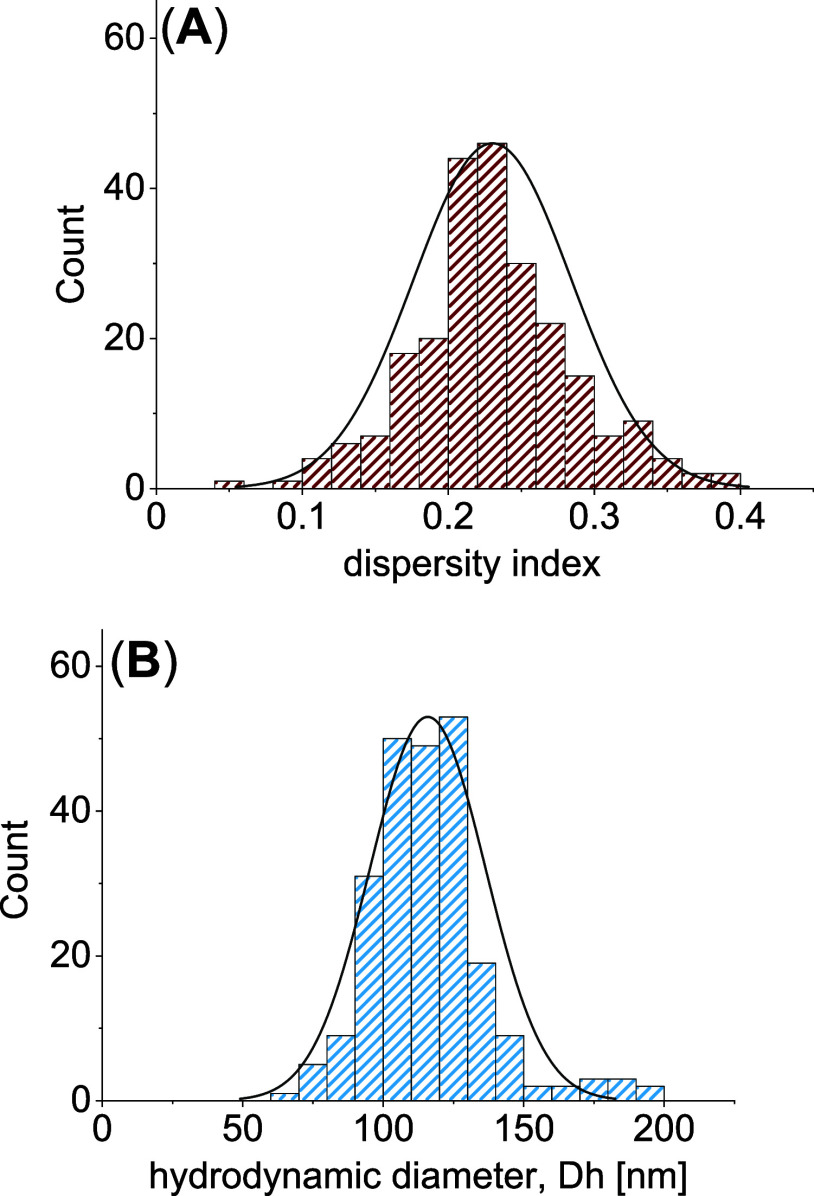
Distribution of monomodal gels in the library by (A) dispersity indices and (B) intensity-weighted hydrodynamic diameters.

The hydrodynamic diameters and dispersities of the synthesized polyacrylates are then determined by dynamic light scattering. Aliquots of each gel are prepared for this analysis by extraction with 1,2-dichloroethane and subsequent dilution with nanopure water as described.^34−36^ The analysis revealed 238 monomodal gels in the library with unique composition with dispersity indices between 0.056 and 0.387 (Supporting Information). However, most monomodal gels have dispersity indices between 0.16 and 0.30 (195 out of 238 gels, Figure 1A), and hydrodynamic diameters between 90 and 140 nm (202 out of 238 gels, Figure 1B). Overall, 46 polyacrylates are classified as nanogels and 192 as microgels. A correlation between the nature, choice, combination, and number of acrylate monomers and the resulting dispersity and sizes of the gels is not apparent, emphasizing the random design of the gel library.

### Gel Screening for Catalytic Hydrolysis of Melibiose

**Scheme 2:**
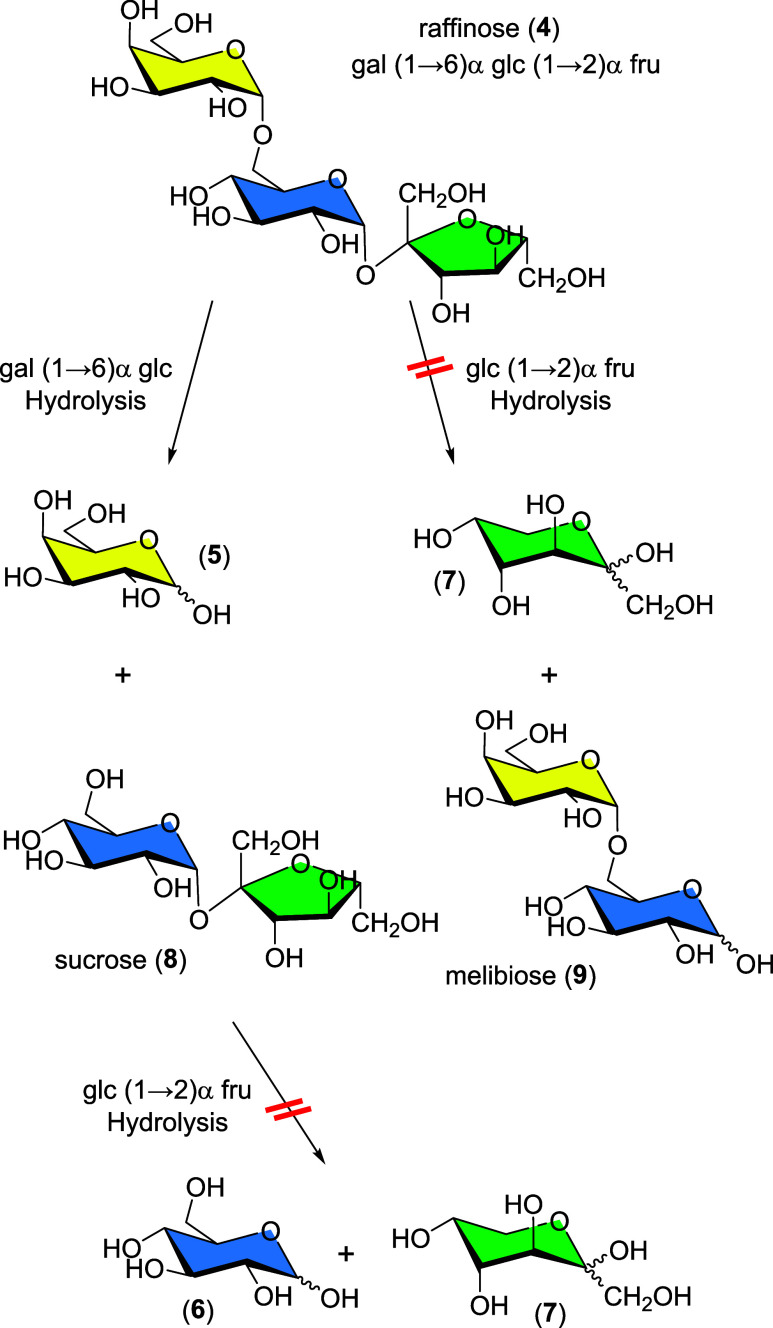
Structure of Raffinose (4) and Putative Saccharides of Its Successive Hydrolysis

Raffinose (**4**) is a nonreducing heterotrisaccharide with two different glycosidic bonds linking three monosaccharides, namely, galactose (**5**), glucose (**6**), and fructose (**7**) (Scheme 2). During the hydrolysis of **4**, mixtures of mono- and disaccharides result that depend on the nature of the released terminal glycosyl moiety. Cleaving off the terminal galactosyl unit (**5**) in **4** by hydrolysis of the 1→6 α-bond results in the formation of sucrose (**8**). By contrast, hydrolyzing the 1→2 α-glycosidic bond causes the release of terminal fructose (**7**) and yields melibiose (**9**). As both, one or none of the glycosidic bonds in **4** may be cleaved by the synthesized gel catalysts, a total of up to 6 different carbohydrates may exist in different proportions in the resulting gel–carbohydrate mixture upon gel-catalyzed hydrolysis of **4**.

Analyzing such a carbohydrate mixture by chromatographic separation, e.g., by HPLC, provides quantitative information about the presence of the respective carbohydrates. However, this procedure has practical limitations when a large library of carbohydrate mixtures is to be analyzed. The largest disadvantage under these circumstances is the time requirement associated with successive chromatographic separations for structurally closely related carbohydrates. An alternative strategy might be found in the simultaneous analysis of a large number of reaction mixtures using a qualitative spectrophotometric assay. This procedure narrows down the number of gels to analyze, focusing on those with the highest potential to cleave the targeted glycosidic bond in a very short amount of time.

In this context, a recently developed combination of a hydrolysis and toluidine assay comes to mind.^34^ While the gel-catalyzed hydrolysis will provide catalyst-dependent mixtures of carbohydrates derived from **4**, the toluidine reagent will not react with ketoses, such as **7** or nonreducing saccharides **4** and **8** eliminating visualization of three of the six potential carbohydrates in the mixture. While targeting the hydrolysis of the 1→6 α-glycosidic bond between the terminal galactosyl and the glucosyl moieties in **4**, only catalysts producing large amounts of **5** are relevant for further analysis and those are identified by a dark green color to the naked eye after the toluidine assay and absorbance reads at Δ*A*_620 nm_ above 0.6 au Even if a catalyst was to cleave the 1→2 α-glycosidic bond in a portion of **4**, the resulting carbohydrates have low absorbance reads at Δ*A*_620 nm_ (Figure S2).^34^ Thus, the design of the assay is suitable for a qualitative analysis of carbohydrate mixtures to identify polyacrylate gels with the highest potential for the catalytic hydrolysis of the 1→6 α-glycosidic bond in the 238-member gel library.

Initially, each monomodal polyacrylate gel of the library is evaluated for its ability to hydrolyze the 1→6 α-glycosidic bond in melibiose (**9**) using the toluidine assay as described.^34,35,37^ In short, the polyacrylate gels are added to 50 mM melibiose solutions and thermostated at 60 °C before the addition of 10 mM aqueous sodium hydroxide solution (Scheme 1).^34,35,37^ After 24 h, an aliquot of the reaction mixture is treated with a 4-fold excess of toluidine reagent and heated to 110–120 °C. After 20 min, the results of the assay are read at 620 nm, and the color distribution over the 96-well plate is preserved by imaging (Figure S1).

The hydrolysis of **9** yields equimolar amounts of glucose (**6**) and galactose (**5**) and both monosaccharides react with the toluidine reagent to form green Schiff bases. The resulting absorbance reads at 620 nm are then significantly higher compared to those of Schiff bases formed from toluidine reagent and **9** or nonreacting control sugars (Figure S2 and Table S1). Gel–carbohydrate mixtures showing absorbance reads above 0.6 au after the toluidine assay are selected for further evaluation toward the hydrolysis of trisaccharide **4** (Table S2). Gels that are unable to hydrolyze **9** as evidenced by a yellow or a yellow-greenish color after the assay and absorbance reads below 0.6 au at 620 nm are thought to have a low potential to cleave the targeted 1→6 α-glycosidic bond and are eliminated from further consideration. The developed assays thereby narrow down a pool of 238 monomodal polyacrylate gels with unique composition to 13 putative catalysts with the potential of hydrolyzing 1→6 α-glycosidic bonds.

### Gel Screening for Catalytic Hydrolysis of Raffinose

**Figure 2:**
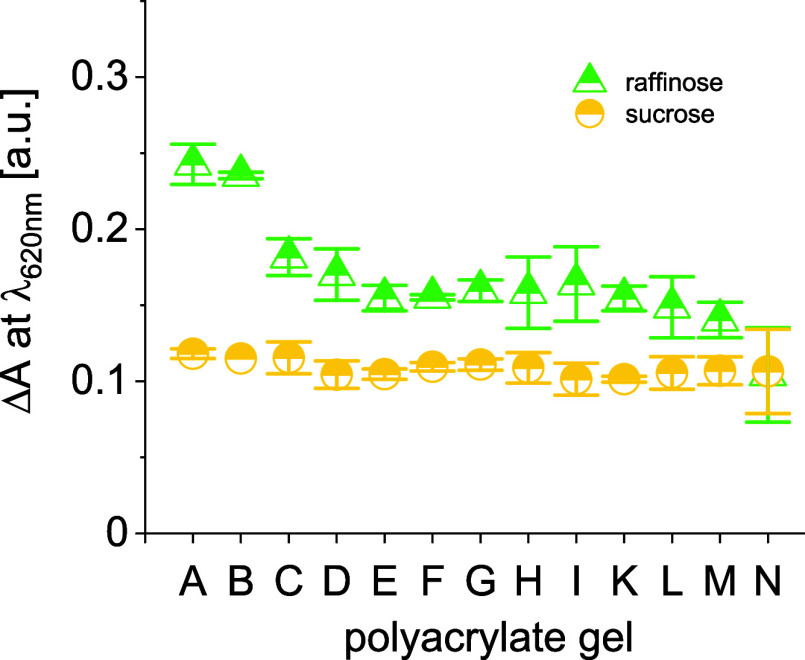
Absorbance reads of Schiff bases formed from hydrolysis assays with selected gels and toluidine reagent.

In a following second step, the 13 potential catalysts (gels A–N, Table S3) are evaluated to estimate their ability to hydrolyze nonreducing trisaccharide **4** and disaccharide **8** using hydrolysis-toluidine assays. Subsequently, the gels are ranked in order of decreasing absorbance reads that are given as an average of 4 independent assays (Figure 2). Notably, the 6 highest ranked gels have each a low amount of 4-hydroxybutyl acrylate (**2g**) in their matrix if any at all (Table S3). Monomer **2g** is the only acrylate among the 7 under investigation to promote H-bond-donating interactions over a hydroxyl group. The finding indicates that the transition state of the hydrolysis of 1→6 α-glycosidic bonds is not primarily stabilized by H-bond donating interactions. Instead, a cocktail of H-bond-accepting and nonpolar monomers with a high content of cyclohexyl acrylate, equal amounts of dodecyl, benzyl, and butyl acrylate, is found efficient for the reaction. Additional correlations between the gel composition and catalytic performance are not apparent at this point. The absorbance reads for the attempted gel-catalyzed hydrolysis of sucrose (**8**) are very steady and within experimental error comparable to the background read of sucrose hydrolysis in the absence of catalyst (Table S1).

The observations indicate that the selected gels do not hydrolyze 1→2 α-glycosidic bonds. The combined results of the toluidine assay analysis suggest selective gel-catalyzed hydrolysis of **4** to release the terminal galactose by hydrolysis of the 1→6 α-glycosidic bond, while complete hydrolysis of **4** by breakage of both glycosidic bonds is rather unlikely (Scheme 1). This feature indicates the selectivity of the gels as found in natural α-galactosidase and renders the gel biomimetic. Control experiments with the remaining 225 gels toward hydrolysis of **4** show absorbance reads of 0.100 ± 0.005 au and an overall similar performance during the hydrolytic assays to those of gel N (Table S2). These observations further underline the initial assumption for their lack of activity toward hydrolyzing the 1→6 α-glycosidic bond. The results further emphasize that gels unable to hydrolyze disaccharide **9** are not able to hydrolyze the same bond in tri- or oligosaccharides. As a control experiment, all other gels in the library are screened toward the hydrolysis of **4** and found inefficient. This conclusion is supported by low absorbance data of the corresponding assay data and their yellow solutions.

### Gel-Catalyzed Hydrolysis of Raffinose

As catalyst sugar mixtures for gels E–M show, within experimental error, comparable absorbance reads after the toluidine assay (Figure 2), gel F is randomly selected as a representative example for this group of gels for further analysis. Additionally, gels A–D and N are selected for evaluation of their ability to cleave the 1→6 α-glycosidic bond in **4** by kinetic studies. Along these lines, aliquots of the selected gels are purified by a sequence of dialysis steps prior to analysis as described.^34,35,37,38^ Unfortunately, aliquots of gel D solidified repetitively during dialysis attempts with aqueous EDTA solution, and therefore, gel D is excluded from further evaluation. Kinetic assays for the catalyzed and uncatalyzed hydrolysis of **4**, **8**, and **9** are subsequently performed and analyzed as described (Table 1).^35−37^

**Table 1:** Kinetic Parameters for the Gel-Catalyzed Hydrolysis of Melibiose (**9**), Raffinose (**4**), and Sucrose (**8**) at 60 °C^a^

entry	gel	monomer composition [mol %]	*k*_cat_ ± Δ*k*_cat_ [min^–1^]	*K*_M_ ± Δ*K*_M_ [mM]	*k*_cat_/*K*_M_ [min^–1^ M^–1^]	*k*_cat_/(*K*_M_ × *k*_non_)
Hydrolysis of Melibiose (**9**)						
1	A	CHA/BA/BnA 25/25/25	0.069 ± 0.013	9.7 ± 0.5	7.1	42,000,000
2	B	DdA/MEA 25/50	0.051 ± 0.007	8.1 ± 0.3	6.3	37,000,000
3	C	MA/CHA/BA/BnA/DdA/HBA 12.5/12.5/12.5/12.5/12.5/12.5	0.018 ± 0.001	7.9 ± 0.1	2.3	14,000,000
4	F	CHA/BnA/MEA 25/25/25	0.002 ± 0.001	7.5 ± 0.6	0.32	1,800,000
5	N	CHA/BA/BnA/DdA/HBA 25/25/12.5/6.25/6.25	0.008 ± 0.001	35 ± 1.7	0.24	1,400,000
Hydrolysis of Raffinose (**4**)						
6	A	CHA/BA/BnA 25/25/25	0.018 ± 0.002	5.6 ± 0.1	3.2	56,000,000
7	B	DdA/MEA 25/50	0.014 ± 0.001	5.6 ± 0.2	2.4	42,000,000
8	C	MA/CHA/BA/BnA/DdA/HBA 12.5/12.5/12.5/12.5/12.5/12.5	0.007 ± 0.001	5.0 ± 0.1	1.4	25,00,000
9	F	CHA/BnA/MEA 25/25/25	0.006 ± 0.001	22.7 ± 0.4	0.27	4,700,000
10	N	CHA/BA/BnA/DdA/HBA 25/25/12.5/6.25/6.25	0.0024 ± 0.0001	10.0 ± 0.3	0.24	4,200,000
Hydrolysis of Sucrose (**8**)						
11	A	CHA/BA/BnA 25/25/25	0.0017 ± 0.0009	7.8 ± 0.9	0.22	28,000

a In the presence of 0.8 mM aqueous NaOH solution; *k*_non_ (**9**) = 1.70 × 10^–7^ min^–1^ M^–1^; *k*_non_ (**4**) = 5.74 × 10^–8^ min^–1^ M^–1^; *k*_non_ (**8**)= 8.0 × 10^–6^ min^–1^ M^–1^.

**Figure 3:**
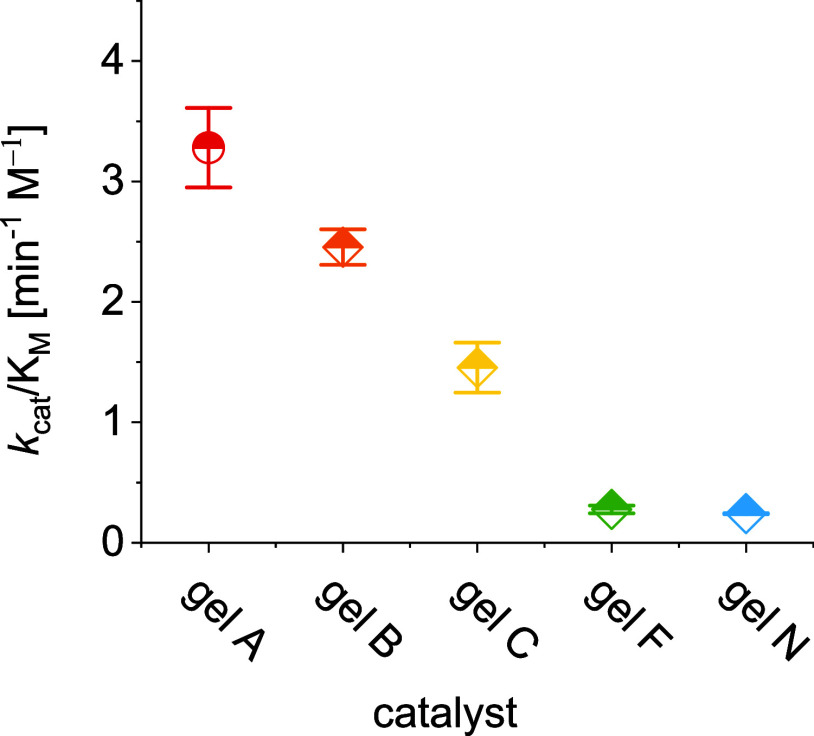
Catalytic efficiency of gels A–C, F, and N for the hydrolysis of raffinose (**4**).

Notably, kinetic parameters for the hydrolysis of **8** are obtained only for gel A with large experimental errors in the assays. A low reaction rate of the hydrolysis and high substrate affinity are observed, demonstrating overall poor catalyst performance for gel A hydrolyzing **8** (Table 1, entry 11). The catalytic efficiency (*k*_cat_/*K*_M_) of the gels in the hydrolysis of **4** is highest for gel A, followed by gels B and C (Figure 3 and Table 1). This order of catalyst performance is the same as that found during the hydrolysis of **9** (Table 1) and during analysis of absorbance reads in toluidine assays for the hydrolysis of the di- and trisaccharide (Figure 2). The three gels show additionally a 10- to 30-fold higher catalytic efficiency than gels F and N upon hydrolysis of **4** (Figure 3). All other gels fail to promote the hydrolysis of **8** indicating that the catalyzed hydrolysis of the 1→2 α-glycosidic bond in **4** is poorly supported by the selected gels.

**Figure 4:**
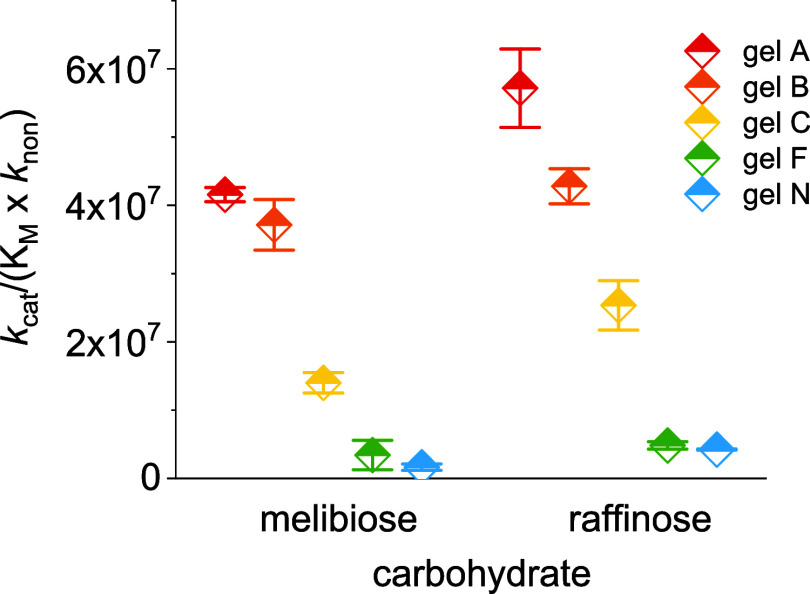
Catalytic proficiency of gels A–C, F, and N for the hydrolysis of melibiose (**9**) and raffinose (**4**).

To compare the performance of the gels toward different substrates, their catalytic proficiency (*k*_cat_/(*K*_M_ × *k*_non_)) accounting for the uncatalyzed background reactions should be used.^39,40^ Notably, the uncatalyzed hydrolysis of disaccharide **9** is about half an order of magnitude faster than the hydrolysis of trisaccharide **4**. Due to this difference, the catalytic proficiency of the gels is slightly higher during hydrolysis of **4** over **9** yet stays in the same order (Table 1 and Figure 4). Given the low probability of all gels to catalyze the hydrolysis of **8**, the combined findings and observations strongly suggest predominant hydrolysis of the targeted 1→6 α-glycosidic bond in **4** and **9** by gels A-C. Overall, gel A has a 1500-fold higher proficiency to hydrolyze the 1→6 α-glycosidic bond in **9** than the 1→2 α-glycosidic bond in **8**. This observation gives another indication that gel A preferably hydrolyzes the 1→6 over the 1→2 α-glycosidic bond in **4**.

The combined observations furthermore suggest that the transition state of the 1→6 α-glycosidic bond is most stabilized when the H-bond accepting interactions of the TEGDMA cross-linker (25 mol %) are supported by equimolar amounts of hydrophobic interaction provided by cyclohexyl (**2b**) and butyl acrylate (**2c**) monomers as well as by CH-π stacking interactions promoted by benzyl acrylate (**2e**) (gel A). The gel-catalyzed hydrolysis of the 1→6 α-glycosidic bond in **4** is also achieved by combining hydrophobic interactions of dodecyl acrylate (**2d**) with H-bond accepting methoxyethyl acrylate (**2f**) in addition to the TEGDMA cross-linker (gel B). Lastly, notable hydrolysis of the 1→6 α-glycosidic bond in **4** is promoted by gels combining equimolar amounts of acrylate monomers **2a**–**e** and **2g** with the H-bond accepting TEGDMA cross-linking backbone. All other gels show poor or insufficient hydrolysis of **4**.

Interestingly, the man-made catalysts show similar contributions to the catalytic turnover through matrix-stabilizing effects, as found in α-galactosidases. A structural and functional analysis of the active site of α-galactosidase from *Saccharomyces cerevisiae* in complex with melibiose and raffinose (3LRL) discloses amino acid residues with a large diversity of side chains.^41^ Aside from the residues of the catalytically active Asp149 and Asp209, various H-bond accepting Arg, Lys, Glu, Asn, Gly, and Asn residues are found in the active site. Additional key contributions are attributed to CH-π stacking interactions of Trp37 and Phe235 along with the hydrophobic interaction of Ala41 to stabilize the galactosyl ring during the turnover of melibiose and raffinose substrates in distorted geometries.^41^

## Conclusions

Initially, a library of 704 polyacrylate gels is synthesized by UV-initiated free radical polymerization of miniemulsions. The prepolymerization mixtures are composed of TEGDMA cross-linker and 7 selected monomers to immobilize a hydrolytic binuclear Cu(II) complex in the presence of a nonionic TWEEN/SPAN surfactant mixture and stabilized by hexadecane as a hydrophobe. Dynamic light scattering determined 238 monomodal gels among the 250 members with unique composition classifying 46 as nanogels and 192 as microgels. A spectrophotometric analysis assay identified 13 gels among those with a high potential to cleave the 1→6 α-glycosidic bond in melibiose (**9**), while their ability to hydrolyze the 1→2 α-glycosidic bond in sucrose (**8**) is low.

Subsequent kinetic assays show a proficiency of 4.2 × 10^7^ for gel A upon hydrolysis of **9** and of 28,000 toward the hydrolysis of **8**. Consequently, gel A is about 1500-fold more proficient to cleave a 1→6 α over a 1→2 α-glycosidic bond in the disaccharides, translating to selective hydrolysis of the glycosidic bonds in raffinose (**4**). The matrix of gel A is composed of 25 mol % TEGDMA cross-linker and equimolar amounts of cyclohexyl, butyl, and benzyl acrylate accounting for nonpolar monomers in the surrounding of the hydrolytic metal complex. The overall catalytic proficiency of gel A toward **4** is 5.6 × 10^7^ rendering gel A among the highest-performing hydrolytic catalysts toward underivatized saccharides and nonactivated glycosidic bonds. Other polyacrylate catalysts (gel B and C) with hydrophobic and H-bond-accepting interactions or equimolar combination of all monomers demonstrate slightly lower ability to hydrolyze 1→6 α-glycosidic bonds in **9**, but failed to provide kinetic data for the hydrolysis of **8**. The observation indicates the strong selectivity of the developed catalysts toward the targeted glycosidic bond.

Several natural and engineered α-galactosidases are reported with a broad range of catalytic performance toward α-glycosidic bonds in natural and unnatural substrates.^41−43^ Catalytic efficiencies toward melibiose (*k*_cat_/*K*_M_ = 2400 M^–1^min^–1;^*k*_cat_ = 0.65 s^–1^; *K*_M_ = 16.9 mM) and raffinose (*k*_cat_/*K*_M_ = 5400 M^–1^min^–1;^*k*_cat_ = 4.2 s^–1^; *K*_M_ = 47.9 mM) hydrolyses are described for α-galactosidase from *Rhizomucor miehei* at pH 4.5 and 50 °C and used here for comparison. Under the assumption that the uncatalyzed hydrolyses of the substrates are comparable to those determined here, the enzymatic hydrolysis of melibiose is 340-fold more proficient than that for gel A and 1700-fold more proficient in the hydrolysis of raffinose than the man-made catalyst. While the enzymatic hydrolysis thus remains unmatched, gel A nevertheless approaches enzyme-like activity. Kinetic data for the α-galactosidase-catalyzed hydrolysis of sucrose are not available. Man-made catalysts with the ability to discriminate selected glycosidic bonds in natural saccharides are also not known at this time, limiting comparison and revealing unprecedented selectivity of gel A for the discrimination of glycosidic bonds.

Overall, the catalytic proficiency of gel A for raffinose and melibiose hydrolysis is 40-fold higher than for gels hydrolyzing turanose,^35^ up to 60-fold higher than for gels hydrolyzing maltose and maltotriose,^34^ and 47-folder higher than for molecularly imprinted polymers hydrolyzing cellobiose (pH 6, 60 °C, *k*_cat_ = 0.027 min^–1^; *K*_M_ = 0.74 mM; *k*_non_ = 3 × 10^–8^ min^–1^ (estimated)).^44^ The combined observations for the catalytic performance of gel A underline a paramount influence of matrix-stabilizing effects on the transition state of hydrolysis of glycosidic bonds. The combined results also provide a rationale for the previously observed inefficiency of gels toward the hydrolysis of 1→6 α-glycosidic bonds using gels that are rationally designed for other purposes. Given the insights into catalyst performance by the spectrophotometric toluidine assays, future attempts for catalyst development can be significantly streamlined.

## Methods and Materials

### Gel Characterization by a Spectrophotometric Screening Assay

All 238 monomodal gels are analyzed for potential catalytic activity with minor modifications of a spectrophotometric toluidine screening assay described recently.^34^ The reagent is prepared as described and stored in a brown glass bottle at −20 °C until use.^34^

### Gel-Catalyzed Hydrolysis of Di- and Oligosaccharides

A 100 μL aliquot of the synthesized gels is diluted 1–4 (v/v) with nanopure water. A 25 μL aliquot of the resulting diluted solutions is added to 180 μL of nanopure water, followed by 25 μL of a 50 mM melibiose or raffinose stock solution. The resulting solution is stirred magnetically and heated to 60 °C for 30–45 min followed by the addition of 20 μL of a 10 mM aqueous sodium hydroxide solution. The resulting solutions are stirred for an additional 24 h, cooled, and stored at −20 °C until analysis.

### Toluidine Assay

Each well in the 96-well plate is filled with sugar-gel solution (25 μL) from the gel-catalyzed hydrolysis of the disaccharide and 100 μL of toluidine reagent. The plate is then heated to 110 °C for 20 min. Subsequently, 75 μL of the resulting solutions are transferred to another plate and the absorbance is immediately read at 620 nm as an end point read at 30 °C. Finally, the plates are scanned to preserve an image of the color distribution as seen by the naked eye.

### Kinetic Hydrolysis Assays and HPLC Methods

The kinetic assays for the preparation of stock solution, hydrolysis, and kinetic analysis assays are performed as described without alteration.^34^ Galactose elutes under the described conditions at 7.1 min, sucrose at 13.0 min, melibiose at 17.2, and raffinose at 26.7 min. The amount of saccharide hydrolyzed and of monosaccharide formed was determined from the observed peak areas by comparison to calibration curves from authentic samples.

## Supporting Information Available

Experimental methods and materials, details for the synthesis of the polyacrylate gel library, dispersity, hydrodynamic diameters determined by DLS, absorbance reads for potential catalysts determined by toluidine-based assays, UV–vis spectra of Schiff bases of selected carbohydrates, details of computation of dipole moments (PDF)
