# Supplementary material for: Selective Hydrolysis of Heterooligosaccharides by Poly(acrylate) Gel Catalysts
Source: ACS Catal. 2024 Oct 30;14(22):16723–30. doi: 10.1021/acscatal.4c04697 (PMC11574753; doi:10.1021/acscatal.4c04697)
Supplement: Supplementary file 1 — cs4c04697_si_001.pdf [file cs4c04697_si_001.pdf]

Selective hydrolysis of heterooligosaccharides by (poly)acrylate gel catalysts

Susanne Striegler*
Department of Chemistry and Biochemistry, 345 North Campus Walk, University of Arkansas, Fayetteville, Arkansas, 72701, United States
Email: Susanne.striegler@uark.edu; phone: +1-479-575-5079; fax: +1-479-575-4049

Content **1**

1 Experimental details **2**

1.1 Instrumentation **2**

1.2 Methods and Materials **2**

1.3 Gel synthesis by radical polymerization in 12-well plates **3**
1.3.1 Stock solutions 3
1.3.2 Preparation of pre-polymerization mixtures and polymerization 4

1.4 Gel characterization by dynamic light scattering **4**
1.4.1 Sample preparation 4
1.4.2 Sample characterization 4

1.5 Gel characterization by a spectrophotometric screening assay **5**
1.5.1 Representative example of a 96-well plate after the toluidine assay (**Figure S1**) 5
1.5.2 UV Vis spectra of Schiff-bases formed from toluidine reagent and selected saccharides over a wavelength range from 400 to 800 nm (**Figure S2, Table S1**) 5
1.5.3 Gel-catalyzed hydrolysis of di- and oligosaccharides (**Table S2, Table S3**) 6
1.5.4 Notes on gel characterizations by gravimetric analysis for determination of the degree of polymerization, substrate accessible surface area, and isothermal titration to determine the metal ion reloading 7

2 Computational analysis data **8**
2.1 Energies, dipole moments, and coordinates of methyl acrylate (2a) **8**
2.2 Energies, dipole moments, and coordinates of cyclohexyl acrylate (2b) **9**
2.3 Summary of dipole moments calculated for monomers used in this study **10**

3 Experimental data **11**
3.1 Monomodal polyacrylate gels from TEGDMA crosslinker: composition, dispersity, and hydrodynamic diameters (Table S4) **11**

4 References **19**

1 Experimental details

1.1 Instrumentation

Intensity-weighted hydrodynamic diameters were obtained on a Malvern Panalytical Zetasizer Nano ZS equipped with a 632.8 nm He-Ne laser, a Malvern Zetasizer NanoSampler, a standard Quartz Flow Cell, and the Malvern Zetasizer Software, version 7.13, for data collection and analysis. Spectrophotometric assays were observed in 96-well plate assays using a FilterMax F5 Multi-Mode Microplate Reader from Molecular Devices in absorbance mode equipped with SoftMax Pro 6 software version 6.3. UV/Vis spectra were recorded at 25 ± 0.1 °C over a range from 400–800 nm on a Varian Cary 50 with WinUV Analysis Suite software, version 3.0. Catalytic hydrolyses of melibiose, sucrose and raffinose were monitored using a HPLC system from Shimadzu equipped with a SCL-10Avp system controller, 2 LC-20AD analytical pumps, DGU-20A3R three channel online degassers, SIL-20A UFLC autosampler with 96 well capability, CTO-20A/ prominence column oven, ELSD-90LT light scattering detector and the LC solution software, version 1.25 for data recording and analysis. A Digital Sonifier (Branson) with flat cap was used for ultrasonication. A medium-pressure lamp TQ 150 (Heraeus Noblelight) with a current of 2.0 A, a voltage of 90 V, and an outer diameter of 13.50 mm was used for free radical polymerizations. Nanopure water at a resistance of 18.2 mΩ was obtained from a ThermoScientific Barnstead E-pure™ water purification system.

1.2 Methods and Materials

For dynamic light scattering, samples were supplied in 2 mL standard clear glass vials for the autosampler supplied with the Malvern Zetasizer instrument; all data were acquired and analyzed using the Malvern Zetasizer Software Version 7.13. Data were recorded using a measuring angle of 173° for back scattering at 20 °C after an equilibration time of 120 s before the first measurement. The data were recorded for settings of the material as a polystyrene latex with a refractive index of 1.590 and nanopure water as the dispersant. Each sample was measured ten times as an average of 10 accumulations each over a 10s duration without delay between measurements. The flow cell is cleaned between measurements three times before acquiring data for additional samples. The reported data are given as an average of the raw data obtained over ten measurements per sample. Absorbance data are recorded at 620 nm at 30 °C as an endpoint read. Clear 96-well polystyrene microplates (Greiner Bio-One) were used for absorbance assays. Heat-resistant polyester films (VWR) were used as 96-well plate adhesives. Absorbance spectra are recorded using a semi-micro quartz cuvette (190–900 nm) with a 1 cm thickness, 10 mm path length and 1 mL volume. The cell is cleaned between measurements by rinsing with 1,3-propanediol. The spectra are given as an average of data recorded from 400-800 nm over three cycles with a scan rate of 400 nm/min, an average time of 0.1 s, and a 0.67 nm data interval. The sugar content in sample aliquots from kinetic assays is monitored using a Luna Amino column (Ø 4.6 × 250 mm) from Phenomenex and 80% acetonitrile as the eluent at a flow rate of 1.0 mL/min at 30 °C. Spectra/Por membranes (Spectrum Labs) with a molecular weight cut-off (MWCO) of 15,000 were used for dialysis. The membranes were soaked in nanopure water for at least 30 min prior to use. The pH values were measured using a Beckman Φ 250 pH meter equipped with a refillable ROSS Orion combination pH electrode with a 165 mm long epoxy body, a 95 mm long semi-micro tip and an 8 mm diameter. The pH meter was calibrated before each set of readings. All buffer solutions were prepared by standard methods at ambient temperature accounting for temperature differences at their intended use.

All chemicals were obtained from commercial suppliers and used as received if not noted otherwise. *N*-cyclohexyl-3-aminopropanesulfonic acid (CAPS), 2,2'-dimethoxy-2-phenylacetophenone, copper(II) acetate monohydrate, sodium hydroxide and D-mannose from Sigma-Aldrich; sucrose (**8**) from Fluka; melibiose (**9**) from Chem-Impex International; 2-methoxyethyl acrylate (**2f**), 4-hydroxybutyl acrylate (**2g**), triethylene glycol dimethacrylate (**1**, TEGDMA), n-hexadecane, TWEEN 80 and SPAN 80 from TCI

America; benzyl acrylate (**2e**), n-dodecyl acrylate (**2d**), methyl acrylate (**2a**) and o-toluidine from Alfa Aesar; 1,3-propanediol, 1,2-dichloroethane, raffinose (**4**), and thiourea from Beantown Chemicals (BTC); butyl acrylate (**2c**), neutral aluminum oxide from Acros Organics; acetic acid and sulfamic acid from EMD Millipore; cyclohexyl acrylate (**2b**) from Thermo Scientific; dimethylsulfoxide from Pharmco-Aarper; and methanol from Supelco. o-Toluidine was distilled in vacuum and stored at –20 °C prior to use in the preparation of the toluidine reagent. All acrylates are purified by filtration over neutral alumina immediately prior to use. The polymerizable ligand *N,N'*-1,3-bis[(2-hydroxy-4-vinylbenzyloxy) benzylideneamino]propan-2-ol (VBbsdpo, **3**) was synthesized as described,[1] mp = 172-175 °C.

1.3 Gel synthesis by radical polymerization in 12-well plates

All gels are synthesized with minor modifications of described procedures.[2-3]

1.3.1 Stock solutions

TWEEN/SPAN/CAPS buffer solutions. Initially, 5 mM CAPS buffer solutions are prepared from 0.5571g in 500mL for use at pH of 10.50 at 0 °C by adjusting the pH with aqueous sodium hydroxide solution to pH 10.08 at 23°C following standard procedures.

For polymerization assays using Cu_2VBbsdpo and TEGDMA crosslinker, aqueous emulsions with an HLB values of 12 are prepared with an overall weight of 200g from 2.43g of SPAN 80 and 6.22g of TWEEN 80 in 5 mM CAPS buffer. The TWEEN 80/SPAN 80 CAPS buffer mixtures are stirred at ambient temperature for 30 min and subsequently sonicated at 40 % amplitude (5 sec on, 2 sec off) for 2 min in the cold. All resulting emulsions are kept at ambient temperature and stirred until use. Typically, 2 mL aliquots of the TWEEN/SPAN/CAPS buffer solution are used during the polymerizations.

Crosslinker/monomer/hydrophobe stock solutions. In a typical procedure, a stock solution of combined 0.3500 mmol of acrylates is prepared consisting of a constant amount of crosslinker (TEGDMA: 25 mol%, 0.0875 mmol, 114.7 µL) and systematically altered amounts of acrylate monomers accounting for 40 mol% of acrylate. The monomer mixtures are derived from butyl acrylate, benzyl acrylate, cyclohexyl acrylate, dodecyl acrylate, methyl acrylate, 4-hydroxybutyl acrylate, and 2-methoxyethyl acrylate. The acrylate monomers are systematically altered to account for 75 mol% of all acrylate content using mixtures of up to 6 of the 7 monomers as 75, 50/25, 25/25/25, 37.5/25/12.5, 25/25/12.5/12.5, 25/25/12.5/6.25/6.25, 12.5/12.5/12.5/12.5/12.5/12.5, 18.75/18.25/12.5/12.5/25/6.25, and 18.75/12.5/12.5/12.5/12.5/6.25 mol%.

For each gel composition, mixtures of 5-times of the needed acrylate amount per well in the 12 well plate are prepared. Finally, 10 µL of hexadecane are added to the acrylate solutions and mixed. The acrylate/hydrophobe mixtures are typically made immediately prior to use and used in two separate aliquots according to the overall volume to synthesize each gel composition in duplicates. The resulting acrylate/hydrophobe mixtures have volumes between 280 and 320 µL, of which aliquots of 55 to 83 µL are used per well.

VBbsdpo stock solution. In a typical experiment, VBbsdpo ligand (116.10 mg, 206.49 µmol) was dissolved in DMSO yielding a solution with a total weight of 1.4401 g that is 8 wt% in ligand. The solution was stored at -20 °C until use, thawed, and used in 11.2 µL aliquots per well.

Cu(II) acetate stock solution. In a typical experiment, copper(II) acetate monohydrate (177.35 mg, 891.25 µmol) was dissolved in 5 mL water, stored at ambient temperature, and used in 20 µL aliquots.

Mannose stock solution. In a typical experiment, mannose (634.80 mg, 3.5267 mmol) was dissolved in 10 mL water, stored at ambient temperature, and used in 50 µL aliquots.

Initiator stock solution. Immediately prior to use, 2,2'-dimethoxyphenylacetophenone (298.80 mg, 1.1658 mmol) is dissolved in 2 × 614.7 µL of methanol, stored in the dark at ambient temperature, and used in 40 µL aliquots.

1.3.2 Preparation of pre-polymerization mixtures and polymerization

In all assays, a total of 0.35 mmol polymerizable monomers and 0.5 mol % of *in-situ* formed polymerizable Cu(II) complex are used for synthesizing gels in 2mL TWEEN 80/ SPAN 80 surfactant mixtures in 12-well plates. In a typical procedure, 12-well plates are filled with 2 mL of the respective TWEEN/SPAN/CAPS buffer emulsions, an aliquot between 55 and 75 µL of crosslinker/monomer/hydrophobe solution depending on monomer composition, followed by 11.2 µL of ligand stock solution, 20 µL of Cu(II) salt stock solution, 50 µL of mannose stock solution, and 40 µL of initiator solution. The mixtures in each well are then sonicated for 30 s each on ice with a sonication horn at 40 % amplitude (5 sec on, 2 sec off). Subsequently, the plates are subjected to UV light in a 15 cm distance to the source in uncovered plates while stirring the miniemulsions in the wells in the cold over 30 min. The resulting gels are subsequently removed from the 12-well plates and stored in 2 mL vessels at ambient temperature until further use.

1.4 Gel characterization by dynamic light scattering (DLS)

All gels are analyzed with by dynamic light scattering as described.[2-3]

1.4.1 Sample preparation

In a typical procedure, a 200 µl aliquot of the synthesized gel dispersion is diluted with 800 µl of nanopure water. The diluted solution was initially extracted with 1,2-dichloroethane (2mL, 3x) and then further diluted by addition of 1000 µL of nanopure water. The resulting solution is further sequentially diluted to 1/1250 and used as such for analysis of the particle size and dispersity index of the gel suspension by dynamic light scattering.

1.4.2 Sample characterization

Data acquisition. All samples were supplied in 2 mL standard autosampler vials. Data for the determination of the mean hydrodynamic diameter were obtained at 20 °C using a measuring angle of 173° for back scattering after an equilibration time of 120 s before the first measurement. The data were recorded for settings of the material as a polystyrene latex with a refractive index of 1.590 and nanopure water as the dispersant. Each sample was measured in duplicate as an average of 10 accumulations each over a 10 s duration time without delay between measurements. The flow cell was cleaned with nanopure water between measurements three times before acquiring data for the next sample.

Data analysis. The reported intensity, volume and number mean data are given as an average of the obtained accumulated measurements per sample.

1.5 Gel characterization by a spectrophotometric screening assay

1.5.1 Representative example of a 96-well plate after the toluidine assay

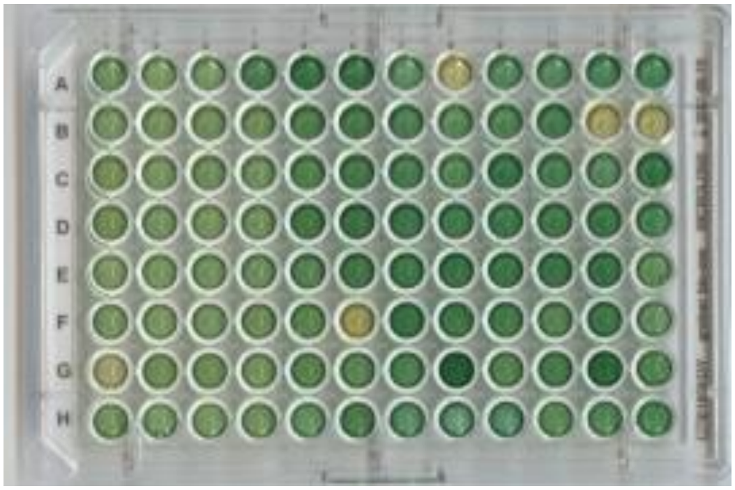

Figure S1. Representative example of a toluidine assay in 96-well plate; each well represents a separate hydrolysis of melibiose with a polyacrylate gel catalyst

1.5.2 UV Vis spectra of Schiff-bases formed from toluidine reagent and selected saccharide standards over a wavelength range from 400 to 800 nm

For measurements of absorbance spectra, 16 wells in two columns of the 96-well plate are filled with 25 µL of 5 mM carbohydrate stock solution followed by 100 µl of toluidine reagent. The mixtures are heated to 110 °C for 20 min and combined. Subsequently, 400 µL of the heated reagent solution are diluted with 600 µL of 1,3-propanediol.

The arbitrary absorbance units of the diluted mixtures are then measured in dependence of the wavelength in three cycles with a scan rate of 400 nm/min and a data interval of 0.67 nm between 400 and 800 nm at 23 °C; the obtained absorbance spectra are given as an average of all measurements. The glucose, galactose and melibiose containing solutions appear green to the naked eye, the solutions containing fructose, sucrose, raffinose and reagent appear yellow.

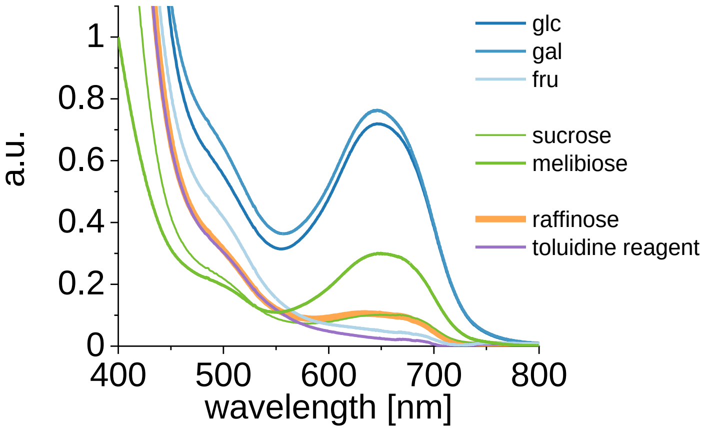

Figure S2. Absorbance reads for selected carbohydrates after heating with toluidine reagent

Table S1. Absorbance reads at 620 nm for Schiff-bases formed from 5 mM carbohydrate standards and toluidine reagent; $\Delta\Delta A_{620}$ = 0.005 a.u.

carbohydrate	ΔA_{620}[a.u.]
Glucose, glc	0.627
Galactose, gal	0.671
Fructose, fru	0.062
sucrose	0.092
melibiose	0.255
raffinose	0.102
toluidine reagent	0.037

1.5.3 Gel-catalyzed hydrolysis of di- and oligosaccharides

Table S2. Absorbance reads of toluidine assays screening with TEGDMA-based polyacrylates for gels with high potential for hydrolysis of melibiose and raffinose; sucrose control*

Gel #	Gel ID	Melibiose $\Delta A_{620} + \Delta\Delta A_{620}$	Raffinose $\Delta A_{620} + \Delta\Delta A_{620}$	Sucrose $\Delta A_{620} + \Delta\Delta A_{620}$
A	921 I05	0.872 ± 0.035	0.243 ± 0.013	0.118 ± 0.003
B	921 D05	0.764 ± 0.067	0.235 ± 0.002	0.115 ± 0.005
C	926 F04	0.846 ± 0.014	0.182 ± 0.012	0.115 ± 0.010
D	926 F10	0.825 ± 0.060	0.170 ± 0.017	0.104 ± 0.009
E	921 J09	0.740 ± 0.074	0.155 ± 0.008	0.105 ± 0.003
F	921 K09	0.969 ± 0.005	0.155 ± 0.002	0.110 ± 0.003
G	926 B11	0.874 ± 0.039	0.160 ± 0.007	0.111 ± 0.004
H	924 I03	0.892 ± 0.013	0.158 ± 0.023	0.109 ± 0.010
I	924 E03	0.690 ± 0.032	0.164 ± 0.024	0.101 ± 0.011
K	921 K11	0.612 ± 0.056	0.155 ± 0.009	0.101 ± 0.002
L	921 I10	0.767 ± 0.098	0.149 ± 0.020	0.106 ± 0.011
M	926 D10	0.825 ± 0.044	0.140 ± 0.012	0.107 ± 0.009
N	924 K04	0.620 ± 0.070	0.104 ± 0.031	0.106 ± 0.028

* The gels are sorted by their absorbance reads for raffinose. All data are given as an average of four independent assays.

Table S3. Monomer composition [mol%] of the gels selected for evaluation of raffinose hydrolysis using toluidine assays; all gels contain 25 mol% TEGDMA crosslinker and 75 mol% monomer for a total of 0.35 mmol combined acrylate content

Gel #	Gel ID	MA [mol %]	CHA [mol %]	BA [mol %]	BnA [mol %]	DdA [mol %]	MEA [mol %]	HBA [mol %]
A	921 I05	--	25	25	25	--	--	--
B	921 D05	--	--	---	--	25	50	--
C	926 F04	12.5	12.5	12.5	12.5	12.5	--	12.5
D	926 F10	12.5	18.75	6.25	12.5	12.5	12.5	--
E	921 J09	25	--	25	--	25	--	--
F	921 K09	--	25	--	25	--	25	--
G	926 B11	12.5	--	25	25	6.25	--	6.25
H	924 I03	25	--	--	25	12.5	--	12.5
I	924 E03	25	25	--	--	--	12.5	12.5
K	921 K11	25	25	--	--	--	25	--
L	921 I10	--	25	25	--	--	--	25
M	926 D10	25	--	25	--	6.25	12.5	6.25
N	924 K04	--	25	25	12.5	6.25	--	6.25

1.5.4 Notes on gel characterizations by gravimetric analysis for determination of the degree of polymerization, substrate accessible surface area, and isothermal titration to determine the metal ion reloading

SPAN 80 and TWEEN 80 are non-dialyzable nonionic surfactants. Thus, once used in the pre-polymerization mixtures, they cannot be removed during the latter dialysis or particle purification. Therefore, elemental analysis data and information about substrate accessible surface area cannot be obtained. Instead, previous extensive efforts to characterize the gel synthesis in 12-well plates showed by gravimetric analysis near quantitative polymerization for each gel studied when the radical polymerization proceeded for 30 min or longer.[3] The elaborated procedure defined the standard polymerization procedure in 12-well plates used here. Separate gravimetric analyses with the pre-polymerization mixtures applied here are not performed.

Likewise, previous efforts showed quantitative reloading of gels with immobilized VBbsdpo ligand by Cu(II) ions by isothermal titration calorimetry. A representative study is published.[4] For all polymers here, quantitative metal ion reloading for all polymers is anticipated based on previous results and used as a foundation for analysis of the kinetic data.

2 Computational analysis data

2.1 Energies, dipole moments, and coordinates of methyl acrylate (2a)

In the gas phase:

Total Energy = -306.467748293 E_H

Standard thermodynamic quantities at 298.180 K and 1.000 ATM:

This Molecule has 0 Imaginary Frequencies

Zero point vibrational energy: 60.093 kcal/mol

Total Enthalpy: 64.853 kcal/mol

Total Entropy: 80.019 cal/mol K

In water:

Total Energy = -306.476376779 E_H

Dipole = 2.049108 D

E_H (LUMO) = -0.04694 E_H

E_H (HOMO) = -0.28171E_H

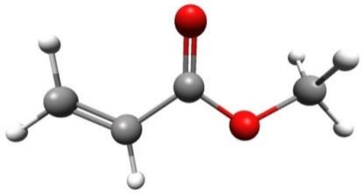

Coordinates (Å)

ATOM	X	Y	Z
C	-1.191682	-2.212674	-0.088090
O	1.225259	-0.672382	0.273295
H	-2.124815	-2.749357	-0.229491
H	-0.298980	-2.799007	0.112619
C	-1.135847	-0.879531	-0.156870
H	-2.015386	-0.274673	-0.356073
C	0.143909	-0.156676	0.038461
H	1.570935	1.838519	1.088659
O	-0.032267	1.174392	-0.077441
C	1.148334	1.985751	0.091763
H	1.896139	1.730388	-0.663006
H	0.814398	3.015250	-0.033827

2.2 Energies, dipole moments, and coordinates of cyclohexyl acrylate (2b)

In the gas phase:

Total Energy = -501.841822709 E_H

Standard thermodynamic quantities at 298.180 K and 1.000 ATM:

This Molecule has 0 Imaginary Frequencies

Zero point vibrational energy:	137.481 kcal/mol
Total Enthalpy:	144.835 kcal/mol
Total Entropy:	102.701 cal/mol K

In water:

Total Energy = -501.850055166 E_H

Dipole = 2.229329 D

E_H (LUMO) = -0.04577 E_H

E_H (HOMO) = -0.27675 E_H

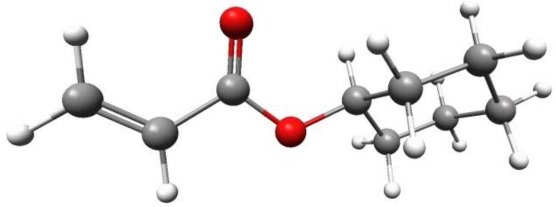

Coordinates (Å)

ATOM	X	Y	Z
C	-0.235213	-3.170019	-1.707352
C	-0.220936	-2.262803	-0.532623
O	-0.173857	-2.622495	0.634062
O	-0.272416	-0.975303	-0.925623
H	-2.053231	0.966571	-0.707485
H	-0.280965	-2.702703	-2.686538
C	-0.193513	-4.496779	-1.555885
H	-0.204662	-5.166006	-2.410804
H	-0.147705	-4.944251	-0.566625
C	-0.271521	0.058202	0.105700
C	1.166599	0.410479	0.491395
C	1.188789	1.588940	1.480604
C	0.433985	2.805849	0.923328
C	-1.001606	2.434350	0.519900
C	-1.022863	1.256422	-0.469517
H	-0.542433	1.554587	-1.411671
H	-0.801341	-0.343702	0.974891
H	1.715880	0.680042	-0.421242
H	1.658388	-0.467990	0.923804
H	0.725552	1.276483	2.427754
H	2.227448	1.853748	1.712550
H	0.969008	3.192056	0.043409
H	0.420471	3.614338	1.664904
H	-1.572981	2.163034	1.419433
H	-1.510877	3.296948	0.073632

2.3 Summary of dipole moments calculated for monomers used in this study.

Energies, dipole moments and coordinates of butyl acrylate (**2c**), 2-methoxyethyl acrylate (**2f**), benzyl acrylate (**2d**), dodecyl acrylate (**2e**), and 4-hydroxybutyl acrylate (**2g**) are calculated and described before.[5-6]

Nonpolar monomers:

- Methyl acrylate (**2a**); dipole = 2.05 D
- Cyclohexyl acrylate (**2b**); dipole = 2.23 D
- Butyl acrylate (**2c**); dipole = 2.25 D[5]
- Benzyl acrylate (**2d**); dipole = 2.48 D[5]
- Dodecyl acrylate (**2e**); dipole = 2.56 D[5]

Polar monomers with H-bond donating and accepting properties:

- 2-methoxyethyl acrylate (**2f**); dipole = 3.74D[5]
- 4-hydroxybutyl acrylate (**2g**); dipole = 4.18 D[6]

3 Experimental data

3.1 Monomodal polyacrylate gels from TEGDMA crosslinker: composition, dispersity, and hydrodynamic diameters (Table S3)

Table S4. Hydrodynamic dynameters and dispersity of gels synthesized from TEGDMA and BA, ChA, MEA, DdA, BnA, MA and HBA

code	Composition [mol %]	PDI	$\overline{X}_{INT} \pm \Delta\overline{X}_{INT}$ [nm]	$\overline{X}_{VOL} \pm \Delta\overline{X}_{VOL}$ [nm]	$\overline{X}_{NUM} \pm \Delta\overline{X}_{NUM}$ [nm]
Monomer mixture [75 mol %]					
Gel 1, 921A01	BA	0.267	132.3 ± 0.1	88.2 ± 1.1	28.8 ± 0.5
Gel 2, 921A04	ChA	0.324	132.8 ± 0.1	91.8 ± 1.5	34.3 ± 1.3
Gel 3, 921A06	MEA	0.251	131.9 ± 2.1	114.3 ± 1.5	56.8 ± 1.2
Gel 4, 921A07	DdA	0.219	160.3 ± 1.4	117.4 ± 1.8	84.5 ± 1.0
Gel 5, 921A09	BnA	0.241	146.4 ± 0.2	137.3 ± 3.2	53.6 ± 3.6
Gel 6, 921A12	MA	0.210	140.1 ± 0.6	139.1 ± 1.1	65.2± 2.9
Gel 7, 924 I05	HBA	0.343	183.1 ± 4.1	139.2 ± 1.5	23.9 ± 5.9
Monomer mixture [50/25 mol %]					
Gel 8, 921B02	BA/ChA	0.226	124.3 ± 0.2	114.2 ± 2.3	55.9 ± 2.1
Gel 9, 921B03	BA/MEA	0.230	118.7 ± 1.1	90.1 ± 1.2	40.3 ± 3.7
Gel 10, 922F06	BA/DdA	0.244	122.6 ± 3.2	88.2 ± 1.4	40.8 ± 1.7
Gel 11, 922F07	BA/BnA	0.146	96.0 ± 2.7	81.2 ± 0.1	57.8 ± 0.5
Gel 12, 922F10	BA/MA	0.246	115.6 ± 3.5	80.2 ± 1.5	31.8 ± 2.2
Gel 13, 922F11	BA/HBA	0.240	111.8 ± 1.1	86.8 ± 2.8	47.1 ± 4.5
Gel 14, 921C01	ChA/BA	0.235	119.9 ± 0.1	86.5 ± 1.3	37.0 ± 1.6
Gel 15, 921C03	ChA/MEA	0.204	105.2 ± 1.6	86.9 ± 1.8	52.0 ± 0.3
Gel 16, 922G06	ChA/DdA	0.154	111.6 ± 1.0	98.7 ± 1.5	63.3 ± 2.0
Gel 17, 921C07	ChA/BnA	0.207	105.9 ± 0.3	87.5 ± 2.4	53.8 ± 0.1
Gel 18, 921C10	ChA/MA	0.222	105.2 ± 1.4	77.7 ± 0.4	41.0 ± 0.3
Gel 19, 921C11	ChA/HBA	0.235	108.8 ± 0.4	85.6± 2.8	45.7 ± 1.0
Gel 20, 921D01	MEA/BA	0.233	110.8 ± 0.1	96.3 ± 2.1	51.5 ± 1.3
Gel 21, 922H04	MEA/ChA	0.165	96.7 ± 2.3	80.7 ± 7.3	25.5 ± 2.0
Gel 22, 921D05	MEA/DdA (gel B)	0.245	124.4 ± 0.1	99.2 ± 1.7	42.8 ± 1.0
Gel 23, 921D07	MEA/BnA	0.209	111.9 ± 0.7	90.3 ± 5.7	47.8 ± 6.5
Gel 24, 922H09	MEA/MA	0.326	157.7 ± 0.4	54.3 ± 1.8	26.1 ± 1.6
Gel 25, 921D12	MEA/HBA	0.239	131.6 ± 1.5	104.8 ± 12.2	43.4 ± 6.3
Gel 26, 921E01	DdA/BA	0.249	115.9 ± 1.0	93.3 ± 5.4	46.6 ± 5.3
Gel 27, 922 I03	DdA/ChA	0.227	119.6 ± 1.6	99.2 ± 1.0	55.1 ± 1.4

code	Composition [mol %]	PDI	$\overline{X}_{INT} \pm \Delta\overline{X}_{INT}$ [nm]	$\overline{X}_{VOL} \pm \Delta\overline{X}_{VOL}$ [nm]	$\overline{X}_{NUM} \pm \Delta\overline{X}_{NUM}$ [nm]
Monomer mixture [50/25 mol %]					
Gel 28, 922 I06	DdA/MEA	0.263	125.2 ± 2.8	102.4 ± 1.4	50.3 ± 1.4
Gel 29, 921E07	DdA/BnA	0.205	128.3 ± 1.6	118.6 ± 1.3	58.9 ± 5.7
Gel 30, 921E10	DdA/MA	0.286	125.3 ± 0.6	111.6 ± 0.1	44.2 ± 3.1
Gel 31, 921E12	DdA/HBA	0.285	121.9 ± 1.6	94.4 ± 1.0	39.6 ± 2.0
Gel 32, 921F02	BnA/BA	0.240	115.6 ± 1.2	58.2 ± 1.3	21.7 ± 3.7
Gel 33, 921F03	BnA/ChA	0.163	119.9 ± 0.4	109.3 ± 0.2	67.0 ± 2.0
Gel 34, 921F06	BnA/MEA	0.199	108.8 ± 0.2	92.8 ± 1.7	55.9 ± 0.6
Gel 35, 921F08	BnA/DdA	0.280	176.1 ± 2.2	137.8 ± 1.5	36.3 ± 7.2
Gel 36, 921F10	BnA/MA	0.204	116.5 ± 0.4	101.6 ± 0.6	65.3 ± 3.6
Gel 37, 922 K11	BnA/HBA	0.235	127.3 ± 0.2	106.3 ± 1.5	51.0 ± 1.8
Gel 38, 921G01	MA/BA	0.253	129.7 ± 0.6	94.5 ± 4.2	45.5 ± 7.4
Gel 39, 921G03	MA/ChA	0.216	120.3 ± 0.4	94.4 ± 2.4	43.4 ± 1.5
Gel 40, 921G06	MA/MEA	0.219	129.3 ± 0.1	112.6 ± 0.5	52.3 ± 1.0
Gel 41, 921G08	MA/DdA	0.259	131.2 ± 0.8	97.4 ± 2.4	54.7 ± 3.0
Gel 42, 921G09	MA/BnA	0.222	129.1 ± 0.4	110.1 ± 0.2	50.4 ± 2.2
Gel 43, 922L11	MA/HBA	0.247	125.8 ± 1.9	82.5 ± 1.2	29.8 ± 0.1
Gel 44, 921H01	HBA/BA	0.210	118.2 ± 0.6	89.0 ± 1.5	35.3 ± 0.5
Gel 45, 921H03	HBA/ChA	0.280	133.6 ± 0.9	92.4 ± 2.4	53.2 ± 3.8
Gel 46, 921H05	HBA/MEA	0.212	104.9 ± 0.4	79.3 ± 1.4	47.3 ± 3.2
Gel 47, 922M07	HBA/DdA	0.224	115.3 ± 1.3	95.1 ± 4.2	48.9 ± 2.3
Gel 48, 921H10	HBA/BnA	0.272	172.9 ± 1.3	147.5 ± 4.3	55.5 ± 2.7
Gel 49, 922 M12	HBA/MA	0.293	142.9 ± 1.0	79.1 ± 2.1	65.2 ± 3.2
Monomer mixture [25/25/25 mol %]					
Gel 50, 921I02	BA/ChA/MEA	0.221	113.0 ± 0.8	87.1 ± 1.4	44.0 ± 0.7
Gel 51, 921I04	BA/ChA/DdA	0.213	120.7 ± 0.8	82.8 ± 2.7	29.6 ± 0.1
Gel 52, 921I05	BA/ChA/BnA (gel A)	0.235	127.6 ± 0.4	97.8 ± 0.6	39.2 ± 1.4
Gel 53, 921I07	BA/ChA/MA	0.234	122.3 ± 0.2	101.2 ± 1.1	53.2 ± 1.5
Gel 54, 921I10	BA/ChA/HBA (gel L)	0.233	125.5 ± 0.1	108.6 ± 0.7	48.5 ± 1.6
Gel 55, 921I11	BA/MEA/DdA	0.247	122.1 ± 0.2	103.8 ± 1.1	47.5 ± 1.3
Gel 56, 921J01	BA/MEA/BnA	0.181	127.2 ± 1.8	122.7 ± 0.5	69.7 ± 0.5
Gel 57, 921J04	BA/MEA/MA	0.221	111.2 ± 0.6	80.7 ± 1.5	37.2 ± 1.2

code	Composition [mol %]	PDI	$\overline{X}_{INT} \pm \Delta\overline{X}_{INT}$ [nm]	$\overline{X}_{VOL} \pm \Delta\overline{X}_{VOL}$ [nm]	$\overline{X}_{NUM} \pm \Delta\overline{X}_{NUM}$ [nm]
Monomer mixture [25/25/25 mol %]					
Gel 58, 921J05	BA/MEA/HBA	0.387	193.3 ± 3.5	191.1 ± 1.5	20.1 ± 0.6
Gel 59, 922O07	BA/DdA/BnA	0.253	133.4 ± 1.8	128.8 ± 1.8	52.7 ± 2.8
Gel 60, 921J09	BA/DdA/MA (gel E)	0.280	113.0 ± 0.1	63.2 ± 1.8	26.8 ± 1.4
Gel 61, 922O11	BA/DdA/HBA	0.215	96.9 ± 1.6	72.7 ± 2.8	42.4 ± 2.6
Gel 62, 921K02	BA/BnA/MA	0.222	108.8 ± 1.3	89.1 ± 0.1	49.0 ± 0.5
Gel 63, 921K04	BA/BnA/HBA	0.208	123.5 ± 0.9	95.2 ± 1.2	38.2 ± 0.8
Gel 64, 921K08	ChA/MEA/DdA	0.227	120.1 ± 0.1	84.2 ± 2.8	37.0 ± 3.7
Gel 65, 921K09	ChA/MEA/BnA (gel F)	0.196	109.2 ± 0.1	91.4 ± 0.2	55.9 ± 1.9
Gel 66, 921K11	ChA/MEA/MA (gel K)	0.207	102.8 ± 1.7	75.2 ± 0.6	54.1 ± 2.8
Gel 67, 923B02	ChA/MEA/HBA	0.218	95.8 ± 0.8	65.7 ± 3.1	38.1 ± 4.0
Gel 68, 923B03	ChA/DdA/BnA	0.235	102.7 ± 0.9	78.9 ± 0.5	44.1 ± 0.1
Gel 69, 921L06	ChA/DdA/MA	0.246	116.7 ± 2.3	84.2 ± 6.8	45.6 ± 1.1
Gel 70, 923B07	ChA/DdA/HBA	0.286	106.7 ± 0.3	99.5 ± 4.7	39.4 ± 1.2
Gel 71, 921L09	ChA/BnA/MA	0.208	109.3 ± 0.1	89.2 ± 4.7	50.1 ± 3.9
Gel 72, 923B11	ChA/BnA/HBA	0.228	97.9 ± 0.1	73.5 ± 1.2	42.0 ± 0.3
Gel 73, 923C02	ChA/MA/HBA	0.214	102.6 ± 0.4	66.3 ± 0.1	26.5 ± 4.2
Gel 74, 921M03	MEA/DdA/BnA	0.242	103.1 ± 1.5	76.4 ± 2.3	42.0 ± 1.9
Gel 75, 923C06	MEA/DdA/MA	0.218	107.7 ± 1.1	72.4 ± 6.5	33.3 ± 5.7
Gel 76, 923C07	MEA/DdA/HBA	0.229	111.6 ± 0.3	76.5 ± 4.8	37.7 ± 1.8
Gel 77, 921M09	MEA/BnA/MA	0.209	96.2 ± 1.9	72.0 ± 2.8	42.7 ± 1.9
Gel 78, 921N02	DdA/BnA/MA	0.187	118.7 ± 0.8	107 ± 1.7	64.3 ± 0.4
Gel 79, 923C11	DdA/BnA/HBA	0.208	104.3 ± 0.1	73.6 ± 2.4	36.0 ± 1.7
Gel 80, 923D05	DdA/MA/HBA	0.250	116.6 ± 0.1	93.2± 1.7	46.5 ± 2.7
Gel 81, 921N07	BnA/MA/HBA	0.237	140.1 ± 0.7	112.2 ± 1.7	77.2 ± 0.9
Monomer mixture [37.5/25/12.5 mol %]					
Gel 82, 923D10	BA/ChA/HBA	0.259	103.0 ± 0.1	93.8 ± 1.7	52.4 ± 1.1
Gel 83, 923D11	BA/MEA/HBA	0.214	110.8 ± 0.3	93.8 ± 1.7	56.7 ± 0.5
Gel 84, 923E01	BA/DdA/HBA	0.207	103.8 ± 0.3	83.1 ± 1.8	47.3 ± 2.2
Gel 85, 923E03	BA/BnA/HBA	0.218	97.8 ± 0.1	77.0 ± 1.4	44.6 ± 2.8
Gel 86, 923E05	BA/MA/HBA	0.233	100.1 ± 0.5	58.1 ± 1.2	53.8 ± 0.9
Gel 87, 922A08	ChA/BA/HBA	0.271	135.1 ± 0.5	80.3 ± 1.3	27.0 ± 1.6

code	Composition [mol %]	PDI	$\overline{X}_{INT} \pm \Delta\overline{X}_{INT}$ [nm]	$\overline{X}_{VOL} \pm \Delta\overline{X}_{VOL}$ [nm]	$\overline{X}_{NUM} \pm \Delta\overline{X}_{NUM}$ [nm]
Monomer mixture [37.5/25/12.5 mol %]					
Gel 88, 923E10	ChA/MEA/HBA	0.258	108.9 ± 1.0	79.0 ± 1.5	43.0 ± 2.1
Gel 89, 922A11	ChA/DdA/HBA	0.264	180.0 ± 0.8	154.1 ± 4.5	34.0 ± 0.3
Gel 90, 923F01	ChA/BnA/HBA	0.163	75.4 ± 0.3	60.0 ± 1.2	44.6 ± 1.2
Gel 91, 923F04	ChA/MA/HBA	0.239	99.6 ± 2.4	63.9 ± 1.1	31.3 ± 3.2
Gel 92, 922B06	MEA/BA/HBA	0.264	127.8 ± 0.3	72.9 ± 0.6	26.2 ± 1.9
Gel 93, 922B08	MEA/ChA/HBA	0.250	116.9 ± 0.3	80.1 ± 4.0	32.9 ± 1.9
Gel 94, 923F10	MEA/DdA/HBA	0.204	122.6 ± 0.7	110.0 ± 1.6	60.7 ± 2.1
Gel 95, 922B11	MEA/BnA/HBA	0.270	127.2 ± 1.4	94.8 ± 4.0	33.8 ± 2.6
Gel 96, 922C01	MEA/MA/HBA	0.194	92.0 ± 1.0	77.2 ± 1.6	52.4 ± 0.4
Gel 97, 922C03	DdA/BA/HBA	0.211	77.3 ± 2.8	73.9 ± 0.4	28.9 ± 3.4
Gel 98, 922C06	DdA/ChA/HBA	0.169	101.8 ± 1.0	87.2 ± 1.6	58.8 ± 1.6
Gel 99, 922C07	DdA/MEA/HBA	0.166	101.4 ± 0.1	85.2 ± 1.0	62.2 ± 0.3
Gel 100, 922C09	DdA/BnA/HBA	0.188	98.1 ± 0.9	68.7 ± 0.5	46.3 ± 0.2
Gel 101, 922C12	DdA/MA/HBA	0.223	123.2 ± 2.7	97.6 ± 2.1	28.7 ± 0.8
Gel 102, 922D01	BnA/BA/HBA	0.114	95.3 ± 0.4	83.5 ± 0.5	62.6 ± 0.7
Gel 103, 922D04	BnA/ChA/HBA	0.111	102.9 ± 0.3	93.0 ± 0.4	70.4 ± 1.3
Gel 104, 922D06	BnA/MEA/HBA	0.108	91.9 ± 1.1	80.8 ± 0.4	62.0 ± 0.9
Gel 105, 922D07	BnA/DdA/HBA	0.176	107.2 ± 1.1	90.5 ± 3.1	54.4 ± 1.8
Gel 106, 922D09	BnA/MA/HBA	0.133	95.8 ± 0.6	83.5 ± 0.6	63.1 ± 0.1
Gel 107, 922D12	MA/BA/HBA	0.218	100.1 ± 1.6	73.1 ± 1.4	42.7 ± 2.4
Gel 108, 922E02	MA/ChA/HBA	0.161	109.4 ± 0.7	95.9 ± 1.3	62.8 ± 0.7
Gel 109, 922E03	MA/MEA/HBA	0.191	102.8 ± 0.1	78.0 ± 2.0	53.9 ± 1.4
Gel 110, 922E06	MA/DdA/HBA	0.201	143.7 ± 1.3	123.3 ± 1.4	88.5 ± 1.0
Gel 111, 922E08	MA/BnA/HBA	0.150	134.7 ± 1.8	129.6 ± 1.4	86.2 ± 0.3
Monomer mixture [25/25/12.5/12.5 mol %]					
Gel 112, 923 I09	BA/ChA/MEA/HBA	0.223	92.3 ± 1.1	62.7 ± 1.3	35.1 ± 2.0
Gel 113, 923 I12	BA/ChA/DdA/HBA	0.248	106.7 ± 3.1	69.6 ± 0.4	26.7 ± 1.6
Gel 114, 923 K02	BA/ChA/BnA/HBA	0.173	84.2 ± 0.7	67.5 ± 1.5	50.8 ± 2.7
Gel 115, 923 K04	BA/ChA/MA/HBA	0.198	81.7 ± 0.5	69.6 ± 1.4	50.6 ± 1.2
Gel 116, 924 A02	BA/DdA/ChA/HBA	0.276	87.2 ± 1.1	69.6 ± 1.2	25.8 ± 1.0
Gel 117, 924 A03	BA/DdA/MEA/HBA	0.261	91.9 ± 0.9	61.0 ± 1.7	34.8 ± 1.6

code	Composition [mol %]	PDI	$\overline{X}_{INT} \pm \Delta\overline{X}_{INT}$ [nm]	$\overline{X}_{VOL} \pm \Delta\overline{X}_{VOL}$ [nm]	$\overline{X}_{NUM} \pm \Delta\overline{X}_{NUM}$ [nm]
Monomer mixture [25/25/12.5/12.5 mol %]					
Gel 118, 924 A06	BA/DdA/BnA/HBA	0.161	95.0 ± 0.7	78.8 ± 0.9	55.7 ± 0.6
Gel 119, 924 A07	BA/DdA/MA/HBA	0.273	111.9 ± 2.2	62.1 ± 1.6	32.5 ± 2.1
Gel 120, 924 A09	BA/BnA/ChA/HBA	0.096	113.0 ± 1.1	105.7 ± 1.0	81.0 ± 0.1
Gel 121, 924 A12	BA/BnA/MEA/HBA	0.229	104.7 ± 0.7	78.8 ± 1.6	45.3 ± 3.7
Gel 122, 924 B01	BA/BnA/DdA/HBA	0.205	104.1 ± 0.8	87.5 ± 1.6	53.8 ± 1.7
Gel 123, 924 B04	BA/BnA/MA/HBA	0.161	99.9 ± 0.7	84.3 ± 0.5	57.3 ± 0.2
Gel 124, 923 K06	BA/MEA/ChA/HBA	0.267	116.3 ± 2.2	107.8 ± 1.6	52.1 ± 2.9
Gel 125, 923 K07	BA/MEA/DdA/HBA	0.351	83.1 ± 2.2	67.8 ± 1.6	36.0 ± 2.8
Gel 126, 924 C02	ChA/MEA/BA/HBA	0.203	115.4 ± 0.6	100.7 ± 1.0	58.9 ± 1.7
Gel 127, 924 C04	ChA/MEA/DdA/HBA	0.250	110.5 ± 0.4	83.7 ± 4.2	42.7 ± 2.5
Gel 128, 924 C05	ChA/MEA/BnA/HBA	0.181	121.1 ± 0.1	109.8 ± 1.4	78.1 ± 1.7
Gel 129, 924 C08	ChA/MEA/MA/HBA	0.218	130.9 ± 0.8	104.5 ± 1.7	55.5 ± 4.2
Gel 130, 924 C09	ChA/DdA/BA/HBA	0.282	128.9 ± 0.3	109.8 ± 1.8	22.8 ± 1.9
Gel 131, 924 C12	ChA/DdA/MEA/HBA	0.331	128.3 ± 0.8	169.9 ± 1.6	31.4 ± 0.7
Gel 132, 924 D02	ChA/DdA/BnA/HBA	0.223	123.6 ± 0.2	109.9 ± 1.1	57.2 ± 2.8
Gel 133, 924 D03	ChA/DdA/MA/HBA	0.257	134.4 ± 1.9	90.0 ± 9.4	31.9 ± 3.0
Gel 134, 924 D06	ChA/BnA/BA/HBA	0.196	120.9 ± 0.1	108.6 ± 1.0	59.8 ± 0.5
Gel 135, 924 D08	ChA/BnA/MEA/HBA	0.201	117.3 ± 0.1	104.8 ± 1.1	60.8 ± 2.9
Gel 136, 924 D09	ChA/BnA/DdA/HBA	0.199	128.0 ± 1.1	116.0 ± 1.1	71.9 ± 0.2
Gel 137, 924 D11	ChA/BnA/MA/HBA	0.218	118.9 ± 0.6	102.7 ± 2.1	57.8 ± 0.5
Gel 138, 924 E02	ChA/MA/BA/HBA	0.220	130.1 ± 0.4	112.6 ± 0.5	56.1 ± 3.6
Gel 139, 924 E03	ChA/MA/MEA/HBA (gel I)	0.222	124.7 ± 0.2	107.7 ± 5.9	55.4 ± 1.4
Gel 140, 924 E05	ChA/MA/DdA/HBA	0.254	148.4 ± 1.6	127.4 ± 4.6	41.2 ± 0.5
Gel 141, 924 E08	ChA/MA/BnA/HBA	0.218	116.6 ± 0.5	81.7 ± 2.8	37.1 ± 2.5
Gel 142, 924 E12	MEA/DdA/ChA/HBA	0.382	120.5 ± 0.3	91.7 ± 1.8	31.7 ± 0.2
Gel 143, 924 F01	MEA/DdA/BnA/HBA	0.199	137.4 ± 0.1	132.1 ± 0.4	69.7 ± 3.4
Gel 144, 924 F04	MEA/DdA/MA/HBA	0.203	131.4 ± 1.9	118.2 ± 0.3	66.6 ± 0.8
Gel 145, 924 F06	MEA/BnA/BA/HBA	0.213	139.0 ± 1.6	122.3 ± 2.6	60.6 ± 1.7
Gel 146, 924 F08	MEA/BnA/ChA/HBA	0.224	122.5 ± 2.1	101.8 ± 0.5	57.1 ± 2.5
Gel 147, 924 F10	MEA/BnA/DdA/HBA	0.301	123.7 ± 0.6	94.8 ± 1.1	21.0 ± 3.1
Gel 148, 924 F11	MEA/BnA/MA/HBA	0.268	119.3 ± 0.2	77.6 ± 0.6	35.6 ± 0.8
Gel 149, 924 G02	MEA/MA/BA/HBA	0.313	192.4 ± 1.5	131± 2.6	102.4 ± 1.1

code	Composition [mol %]	PDI	$\overline{X}_{INT} \pm \Delta\overline{X}_{INT}$ [nm]	$\overline{X}_{VOL} \pm \Delta\overline{X}_{VOL}$ [nm]	$\overline{X}_{NUM} \pm \Delta\overline{X}_{NUM}$ [nm]
Monomer mixture [25/25/12.5/12.5 mol %]					
Gel 150, 924G04	MEA/MA/ChA/HBA	0.250	170.3 ± 0.8	125.3 ± 2.1	64.5 ± 3.7
Gel 151, 924G05	MEA/MA/DdA/HBA	0.341	180.1 ± 1.3	155 ± 1.9	39.5 ± 0.2
Gel 152, 924G07	MEA/MA/BnA/HBA	0.330	124.2 ± 2.6	110.5 ± 1.6	43.0 ± 1.6
Gel 153, 924G09	DdA/BnA/BA/HBA	0.219	119.4 ± 0.1	91.8 ± 1.8	36.6 ± 8.6
Gel 154, 924G11	DdA/BnA/ChA/HBA	0.199	125.9 ± 0.4	114.7 ± 6.1	64.8 ± 7.2
Gel 155, 924H01	DdA/BnA/MEA/HBA	0.271	125.1 ± 2.2	77.6 ± 0.8	29.1 ± 2.7
Gel 156, 924H03	DdA/BnA/MA/HBA	0.244	118.9 ± 1.3	96.5 ± 0.8	50.3 ± 1.2
Gel 157, 924H05	DdA/MA/BA/HBA	0.269	120.9 ± 1.2	92.3 ± 0.8	43.8 ± 0.8
Gel 158, 924H07	DdA/MA/ChA/HBA	0.275	164.9 ± 1.1	135.4 ± 1.6	38.6 ± 2.5
Gel 159, 924H10	DdA/MA/MEA/HBA	0.329	125.8 ± 0.1	96.5 ± 1.8	61.3 ± 3.2
Gel 160, 924H11	DdA/MA/BnA/HBA	0.235	118.8 ± 0.8	95.6 ± 1.8	46.7 ± 2.2
Gel 161, 922E09	BnA/MA/BA/HBA	0.209	86.6 ± 2.0	65.1 ± 1.2	43.5 ± 1.5
Gel 162, 922E11	BnA/MA/ChA/HBA	0.131	98.2 ± 0.6	85.3 ± 0.6	62.3 ± 0.7
Gel 163, 924I01	BnA/MA/MEA/HBA	0.225	126.3 ± 2.6	108.7 ± 5.8	57.2 ± 4.6
Gel 164, 924I03	BnA/MA/DdA/HBA (gel H)	0.250	127.7 ± 1.9	85.0 ± 2.7	32.7 ± 3.9
Monomer mixture [25/25/12.5/6.25/6.25 mol %]					
Gel 165, 924I07	BA/CHA/DdA/MEA/HBA	0.175	85.3 ± 0.8	66.5 ± 2.7	44.3 ± 3.8
Gel 166, 924I09	BA/CHA/BnA/MEA/HBA	0.056	111.6 ± 0.5	106.2 ± 0.5	88.7 ± 1.1
Gel 167, 924I11	BA/CHA/MA/MEA/HBA	0.100	103.3 ± 0.6	93.1 ± 2.1	70.2 ± 5.8
Gel 168, 924K01	BA/CHA/MEA/DdA/HBA	0.218	100.8 ± 2.1	74.8 ± 1.5	52.4 ± 1.7
Gel 169, 924K04	BA/CHA/BnA/DdA/HBA (gel N)	0.121	99.0 ± 0.9	86.5 ± 1.4	63.1 ± 2.0
Gel 170, 924K05	BA/CHA/MA/DdA/HBA	0.154	104.0 ± 0.6	90.5 ± 1.8	64.9 ± 2.4
Gel 171, 924K07	BA/CHA/MEA/BnA/HBA	0.148	100.0 ± 1.2	85.6 ± 1.0	60.5 ± 5.0
Gel 172, 924K09	BA/CHA/DdA/BnA/HBA	0.211	90.1 ± 1.2	66.3 ± 1.0	42.8 ± 0.8
Gel 173, 924K12	BA/CHA/MA/BnA/HBA	0.166	106.8 ± 0.3	88.0 ± 0.3	51.8 ± 1.3
Gel 174, 924L02	BA/CHA/MEA/MA/HBA	0.154	101.3 ± 0.8	87.0 ± 1.0	63.7 ± 0.6
Gel 175, 924L04	BA/CHA/DdA/MA/HBA	0.271	103.0 ± 2.4	111.0 ± 2.8	31.0 ± 3.1
Gel 176, 924L06	BA/CHA/BnA/MA/HBA	0.139	100.3 ± 0.8	87.2 ± 1.6	62.9 ± 2.0
Gel 177, 924L08	BA/MEA/DdA/ChA/HBA	0.241	96.9 ± 0.3	53.2 ± 0.9	25.0 ± 3.1
Gel 178, 924L09	BA/MEA/BnA/ChA/HBA	0.164	89.3 ± 2.0	71.9 ± 0.5	50.3 ± 3.3
Gel 179, 924L11	BA/MEA/BnA/MA/HBA	0.208	113.0 ± 0.4	95.0 ± 1.2	54.7 ± 3.2

code	Composition [mol %]	PDI	$\overline{X}_{INT} \pm \Delta\overline{X}_{INT}$ [nm]	$\overline{X}_{VOL} \pm \Delta\overline{X}_{VOL}$ [nm]	$\overline{X}_{NUM} \pm \Delta\overline{X}_{NUM}$ [nm]
Monomer mixture [25/25/12.5/6.25/6.25 mol %]					
Gel 180, 924 M01	BA/MEA/ChA/DdA/HBA	0.179	99.7 ± 1.1	81.3 ± 1.5	53.8 ± 2.1
Gel 181, 924 M03	BA/MEA/BnA/DdA/HBA	0.214	102.7 ± 1.1	81.2 ± 0.2	48.9 ± 1.3
Gel 182, 924 M05	BA/MEA/MA/DdA/HBA	0.235	98.7 ± 0.3	63.7 ± 1.0	50.5 ± 1.9
Gel 183, 924 M08	BA/MEA/ChA/BnA/HBA	0.165	112.6 ± 1.0	99.1 ± 1.0	68.4 ± 1.0
Gel 184, 924 M10	BA/MEA/DdA/BnA/HBA	0.163	115.1 ± 1.8	103.7 ± 2.6	70.4 ± 1.7
Gel 185, 924 M11	BA/MEA/MA/BnA/HBA	0.191	120.1 ± 0.2	101.9 ± 0.5	55.0 ± 1.2
Gel 186, 924 N02	BA/MEA/ChA/MA/HBA	0.240	133.2 ± 0.5	53.7 ± 1.5	39.6± 0.2
Gel 187, 924 N04	BA/MEA/DdA/MA/HBA	0.282	106.5 ± 0.4	63.5 ± 1.3	31.5 ± 2.4
Gel 188, 924 N05	BA/MEA/BnA/MA/HBA	0.231	101.1± 5.9	32.8 ± 1.4	24.2 ± 1.4
Gel 189, 924 N07	BA/DdA/MEA/ChA/HBA	0.277	140.4 ± 2.8	90.3 ± 2.1	31.2 ± 2.0
Gel 190, 924 N09	BA/DdA/BnA/ChA/HBA	0.366	61.2 ± 0.4	49.8 ± 1.5	25.0 ± 0.2
Gel 191, 924 N11	BA/DdA/MA/ChA/HBA	0.364	70.9 ± 0.5	64.6 ± 2.1	24.2 ± 0.4
Gel 192, 924 O01	BA/DdA/ChA/MEA/HBA	0.199	99.1 ± 0.3	79.5 ± 1.1	49.3 ± 0.2
Gel 193, 924 O04	BA/DdA/BnA/MEA/HBA	0.195	104.5 ± 1.1	88.3 ± 1.6	57.5 ± 2.1
Gel 194, 924 O05	BA/DdA/BnA/MA/HBA	0.134	124.7 ± 0.1	117.3 ± 0.9	75.9 ± 2.2
Gel 195, 924 O07	BA/DdA/ChA/BnA/HBA	0.163	112.2 ± 0.1	100.0 ± 0.3	64.6 ± 0.3
Gel 196, 924 O10	BA/DdA/MEA/BnA/HBA	0.234	83.4 ± 0.1	50.9 ± 0.3	28.5 ± 0.6
Gel 197, 924 O11	BA/DdA/MA/BnA/HBA	0.256	71.7 ± 0.3	42.5 ± 2.9	30.6 ± 0.9
Gel 198, 926A02	BA/DdA/CHA/MA/HBA	0.284	97.7 ± 2.0	133.2 ± 0.1	16.0 ± 5.6
Gel 199, 926A04	BA/DdA/MEA/MA/HBA	0.322	111.4 ± 0.5	97.0 ± 2.8	20.9 ± 1.5
Gel 200, 926A05	BA/DdA/BnA/MA/HBA	0.308	102.2 ± 0.9	98.6 ± 3.9	51.1 ± 0.1
Gel 201, 926A07	BA/BnA/MEA/ChA/HBA	0.152	90.1 ± 0.5	73.5 ± 1.7	51.4 ± 3.0
Gel 202, 926A10	BA/BnA/DdA/ChA/HBA	0.322	93.3 ± 3.1	86.8 ± 1.9	24.3 ± 5.2
Gel 203, 926A12	BA/BnA/MA/ChA/HBA	0.122	77.5 ± 0.2	65.3 ± 0.5	50.2 ± 0.8
Gel 204, 926B02	BA/BnA/ChA/MEA/HBA	0.184	81.2 ± 0.8	61.7 ± 0.9	43.9 ± 1.4
Gel 205, 926B03	BA/BnA/DdA/MEA/HBA	0.194	99.2 ± 1.5	82.5 ± 1.1	57.4 ± 0.6
Gel 206. 926B06	BA/BnA/MA/MEA/HBA	0.234	98.8 ± 1.1	74.8 ± 1.5	53.5 ± 0.2
Gel 207, 926B07	BA/BnA/ChA/DdA/HBA	0.168	102.8 ± 0.2	88.4 ± 0.4	62.1 ± 0.4
Gel 208, 926B10	BA/BnA/MEA/DdA/HBA	0.191	93.3 ± 0.3	73.6 ± 2.6	52.6 ± 5.0
Gel 209, 926B11	BA/BnA/MA/DdA/HBA (gel G)	0.181	94.0 ± 0.3	75.4 ± 1.7	50.1 ± 2.5
Gel 210, 926C02	BA/BnA/ChA/MA/HBA	0.218	100.6 ± 0.5	74.7 ± 1.5	45.4 ± 3.3
Gel 211, 926C03	BA/BnA/MEA/MA/HBA	0.266	103.5 ± 1.5	61.1 ± 0.5	27.5 ± 0.1

code	Composition [mol %]	PDI	$\overline{X}_{INT} \pm \Delta\overline{X}_{INT}$ [nm]	$\overline{X}_{VOL} \pm \Delta\overline{X}_{VOL}$ [nm]	$\overline{X}_{NUM} \pm \Delta\overline{X}_{NUM}$ [nm]
Monomer mixture [25/25/12.5/6.25/6.25 mol %]					
Gel 212, 926C06	BA/BnA/DdA/MA/HBA	0.327	131.2 ± 3.2	75.8 ± 1.5	58.6 ± 0.3
Gel 213, 926C07	BA/MA/MEA/ChA/HBA	0.227	128.6 ± 1.7	78.8 ± 1.3	49.8 ± 1.5
Gel 214, 926C10	BA/MA/DdA/ChA/HBA	0.238	118.6 ± 1.3	75.1 ± 0.9	42.4 ± 0.7
Gel 215, 926C11	BA/MA/BnA/ChA/HBA	0.223	112.6 ± 0.9	75.3 ± 2.0	41.5 ± 3.7
Gel 216, 926D02	BA/MA/ChA/MEA/HBA	0.264	124.3 ± 1.6	74.7 ± 1.5	51.5 ± 1.2
Gel 217, 926D03	BA/MA/DdA/MEA/HBA	0.296	142.5 ± 0.7	78.8 ± 1.2	65.5 ± 0.7
Gel 218, 926D05	BA/MA/BnA/MEA/HBA	0.228	127.6 ± 2.5	71.1 ± 0.8	47.7 ± 0.4
Gel 219, 926D07	BA/MA/ChA/DdA/HBA	0.311	115.8 ± 1.5	73.3 ± 2.0	54.8 ± 0.4
Gel 220, 926D10	BA/MA/MEA/DdA/HBA (gel M)	0.311	107.5 ± 1.6	88.8 ± 1.5	47.8 ± 0.1
Gel 221, 926D12	BA/MA/BnA/DdA/HBA	0.344	109.2 ± 2.5	75.1 ± 1.8	47.1 ± 1.2
Gel 222, 926E02	BA/MA/ChA/BnA/HBA	0.280	121.1 ± 0.2	78.3 ± 2.0	38.5 ± 1.9
Gel 223, 926E03	BA/MA/MEA/BnA/HBA	0.266	101.8 ± 0.9	82.8 ± 1.4	47.3 ± 1.2
Gel 224, 926E06	BA/MA/DdA/BnA/HBA	0.317	131.9 ± 2.1	76.1 ± 0.8	60.1 ± 0.5
Monomer mixture [12.5/12.5/12.5/12.5/12.5/12.5 mol %]					
Gel 225, 926E07	BA/ChA/MEA/DdA/BnA/MA	0.237	155.1 ± 0.1	72.1 ± 0.8	95.5 ± 5.9
Gel 226, 926E09	BA/ChA/MEA/DdA/BnA/HBA	0.284	136.9 ± 1.5	73.3 ± 2.0	77.2 ± 0.3
Gel 227, 926E12	BA/ChA/MEA/DdA/HBA/MA	0.275	145.9 ± 5.7	86.8 ± 1.6	51.3 ± 3.6
Gel 228, 926F02	BA/ChA/MEA/BnA/MA/HBA	0.254	125.6 ± 0.8	74.1 ± 1.9	73.7 ± 0.2
Gel 229, 926F04	BA/ChA/DdA/BnA/MA/HBA (gel C)	0.296	116.3 ± 0.5	77.3 ± 2.0	56.7 ± 0.1
Gel 230, 926F05	BA/MEA/DdA/BnA/MA/HBA	0.275	125.6 ± 1.0	78.3 ± 2.3	48.0 ± 1.6
Gel 231, 926F07	ChA/MEA/DdA/BnA/MA/HBA	0.216	129.4 ± 0.8	92.2 ± 3.3	25.6 ± 0.7
Monomer [18.75/18.25/12.5/12.5/6.25/6.25 mol %]					
Gel 232, 926F12	ChA/BnA/MEA/DdA/BA/HBA	0.233	117.2 ± 0.1	86.8 ± 2.8	43.1 ± 3.5
Gel 233, 926G01	ChA/DdA/MEA/MA/BA/HBA	0.284	113.3 ± 2.1	75.3 ± 2.1	34.8 ± 2.3
Gel 234, 926G03	ChA/BnA/MEA/MA/BA/HBA	0.301	125.9 ± 1.1	74.3 ± 2.2	66.8 ± 4.3
Gel 235, 926G06	ChA/BnA/DdA/MA/BA/HBA	0.236	125.3 ± 1.6	104.7 ± 4.1	48.7 ± 2.5
Gel 236, 926G07	DdA/BnA/MEA/MA/BA/HBA	0.231	116.9 ± 0.2	81.7 ± 7.8	37.1 ± 7.1
Monomer [18.75/12.5/12.5/12.5/12.5/6.25 mol %]					
Gel 237, 926F10	ChA/MEA/DdA/BnA/MA/BA (gel D)	0.337	118.3 ± 1.9	81.7 ± 2.8	53.4 ± 3.9
Gel 238, 926G09	BnA/CHA/MEA/DdA/MA/HBA	0.229	104.8 ± 1.3	83.3 ± 1.8	48.7 ± 1.4

4 References

1. Striegler, S.; Dunaway, N. A.; Gichinga, M. G.; Barnett, J. D.; Nelson, A.-G. D., Evaluating Binuclear Copper(II) Complexes for Glycoside Hydrolysis *Inorg. Chem.* **2010,** 49 (6), 2639-2648.
2. Striegler, S., Developing catalysts for the hydrolysis of glycosidic bonds in oligosaccharides using a spectrophotometric screening assay *ACS Catal.* **2024,** 14 (17), 12940–12946.
3. Sharma, B.; Striegler, S., Nanogel Catalysts for the Hydrolysis of Underivatized Disaccharides Identified by a Fast Screening Assay *ACS Catal.* **2023,** 13 (3), 1614-1620.
4. Sharma, B.; Striegler, S., Nonionic Surfactant Blends to Control the Size of Microgels and Their Catalytic Performance during Glycoside Hydrolyses *ACS Catal.* **2020,** 10 (16), 9458-9463.
5. Sharma, B.; Striegler, S., Tailored Interactions of the Secondary Coordination Sphere Enhance the Hydrolytic Activity of Cross-Linked Microgels *ACS Catal.* **2019,** 9 (3), 1686-1691.
6. Clem, C. M.; Sharma, B.; Striegler, S., Structure-activity-relationship studies to elucidate sources of antibacterial activity of modular polyacrylate microgels *ACS Appl. Bio Mater.* **2021,** 4 (10), 7578–7586.
